# *Streptococcus pneumoniae* HtrA is a dynamic and monomeric virulence factor capable of forming larger oligomeric complexes

**DOI:** 10.1002/pro.70411

**Published:** 2025-12-28

**Authors:** Eunjeong Lee, Jasmina S. Redzic, Blaine Gordon, Anthony J. Saviola, Norman Tran, Sean P. Maroney, Nathanael L. Ashby, Steven Shaw, Sam Fulte, Arianna McCarty, Todd Holyoak, Nancy Meyer, Kirk C. Hansen, Sarah E. Clark, Elan Eisenmesser

**Affiliations:** ^1^ Department of Biochemistry and Molecular Genetics, School of Medicine University of Colorado Anschutz Medical Campus, School of Medicine Aurora Colorado USA; ^2^ Department of Biology University of Waterloo Waterloo Ontario Canada; ^3^ Department of Otolaryngology – Head & Neck Surgery, School of Medicine University of Colorado Anschutz Medical Campus, School of Medicine Aurora Colorado USA; ^4^ Pacific Northwest Cryo‐EM Center Oregon Health and Science University Portland Oregon USA

**Keywords:** cryo‐EM, Gram‐positive, HtrA, infection, NMR, structure, virulence factor

## Abstract

High‐temperature requirement A (HtrA) proteases are a conserved family of serine proteases central to protein quality control and bacterial virulence. While Gram‐negative and human HtrAs are structurally well studied, Gram‐positive homologs remain essentially uncharacterized. Here, we present the first integrated structural and mechanistic analysis of a Gram‐positive HtrA, from *Streptococcus pneumoniae*, a virulence factor essential for adhesion and infection in vivo. Proteomic profiling of an *htrA* knockout and cleavage assays demonstrate that *S. pneumoniae* HtrA is required for protein quality control, with the PDZ domain mediating substrate recognition. Biochemically, *S. pneumoniae* HtrA exists exclusively as a monomer in solution, a striking divergence from canonical trimeric HtrAs that we show is shared with other Gram‐positive homologs. NMR analyses reveal that the monomer dynamically samples open and closed conformations, while cryo‐EM of a catalytic mutant identifies a hexamer stabilized by a unique LoopA–PDZ interaction. Together, these findings define *S. pneumoniae* HtrA as a dynamic monomer with interdomain coupling between its protease and PDZ domains, establishing Gram‐positive HtrAs as a mechanistically divergent subgroup within the HtrA family.

## 1 INTRODUCTION

*Streptococcus pneumoniae* (*S. pneumoniae*) is a leading cause of global morbidity and mortality, responsible for diseases including pneumonia, sepsis, and meningitis. The World Health Organization (WHO) has designated *S. pneumoniae* a “high priority” pathogen due to its disease burden and growing resistance to existing treatments (Ramirez et al., 2017). Current vaccines target capsular polysaccharides but are limited by the emergence of non‐vaccine serotypes (Kim et al., 2017), prompting efforts to identify conserved protein‐based vaccine or therapeutic targets (Breijyeh et al., 2020; Ramirez et al., 2017). One such candidate is the high‐temperature requirement A (HtrA) serine protease, a multifunctional protein implicated in bacterial protein quality control, virulence, and immune evasion.

**FIGURE 1:**
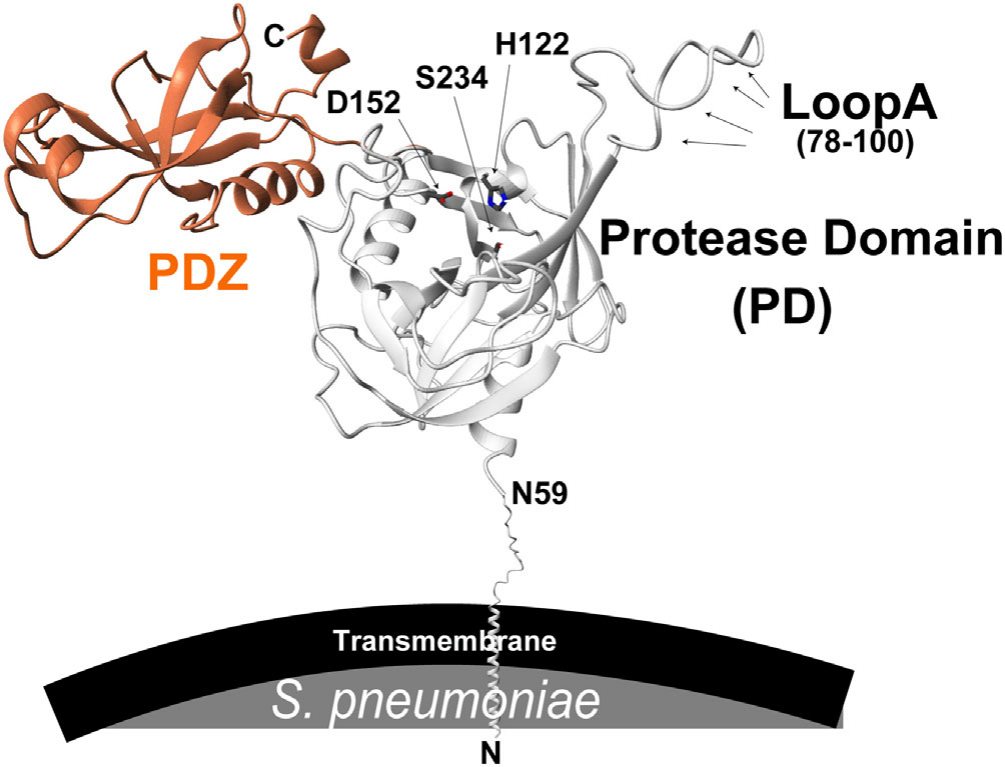
*S. pneumoniae* HtrA. AlphaFold predicted model of HtrA with key elements shown, including the N‐terminal PD and the C‐terminal PDZ. LoopA and the catalytic triad are also delineated. Like most HtrAs, *S. pneumoniae* HtrA is initially tethered to the membrane through an N‐terminal transmembrane region.

HtrA proteases are typically membrane‐associated via an N‐terminal transmembrane helix but family members, including that from *S. pneumoniae*, can also be released as soluble proteins following signal peptide cleavage (Figure 1) (Ali et al., 2021; Backert et al., 2018; Song et al., 2022). In several bacterial pathogens—including *Escherichia coli* (*E. coli*), *Yersinia enterocolitica*, *Helicobacter pylori* (*H. pylori*), *Listeria monocytogenes*, and *S. pneumoniae—HtrA* plays an important role in infection by proteolyzing misfolded proteins (Backert et al., 2018; de Stoppelaar et al., 2013; Heusipp et al., 2004; Radhakrishnan et al., 2021; Zhang et al., 2019). However, while Gram‐negative HtrAs have been structurally and biochemically characterized, much less is known about the mechanisms underlying HtrA function in Gram‐positive bacteria like *S. pneumoniae*. Our studies here are the first to probe multiple Gram‐positive bacteria HtrA enzymes.

A hallmark of most HtrA proteases—spanning Gram‐negative bacteria and humans—is the formation of a conserved trimeric core composed of protease domains (PDs) and at least one C‐terminal PDZ domain. In Gram‐negative bacteria, DegS‐type HtrAs contain one PDZ domain that forms trimers, whereas DegP‐ and DegQ‐type HtrAs contain two PDZ domains that often form larger oligomeric complexes with trimers as the basic unit (Krojer et al., 2010; Wilken et al., 2004). Human HtrAs all contain a single PDZ domain that assembles into trimers but can also form larger oligomers (Eigenbrot et al., 2012; Li et al., 2002). These trimeric cores serve as platforms for regulated proteolysis and can further organize into hexamers, dodecamers, or even 24‐mers in response to specific signals or substrates (Malet et al., 2012; Sawa et al., 2011). Notably, structural analysis of human HtrA2 revealed a closed conformation in which the PDZ domain sterically occludes the PD active site, suggesting that dynamic transitions between open and closed states may regulate protease activity (Li et al., 2002). However, this dynamic behavior has been difficult to directly observe using solution‐based methods like NMR, due to the large size of these oligomers.

Some bacterial HtrAs also exhibit pH‐sensitive transitions between oligomeric states. For example, *H. pylori* HtrA dissociates into monomers at acidic pH (Cui et al., 2023) and *Mycobacterium tuberculosis* (*M. tuberculosis*) PepD samples a monomer–trimer equilibrium under physiological conditions (Gupta et al., 2018). However, these monomeric forms are in equilibrium with trimeric forms where both oligomeric states are observed simultaneously. This framework sets the stage for evaluating whether Gram‐positive HtrAs share similar mechanisms or diverge entirely.

Despite advances in the HtrA field, structural insights into Gram‐positive HtrA family members remain limited—even though they play crucial roles in bacterial virulence. *S. pneumoniae* HtrA has been shown to be essential for infection in murine models (de Stoppelaar et al., 2013), yet its structural and biochemical properties are largely undefined. Like other HtrA proteases, *S. pneumoniae* HtrA comprises a PD with a conserved catalytic triad (H122–D152–S234) (Figure 1). However, unlike its well‐characterized Gram‐negative counterparts, Gram‐positive HtrAs such as *S. pneumoniae* HtrA must function in a cell envelope lacking an outer membrane, raising fundamental questions about its biological roles, regulatory mechanisms, and oligomeric state. Clarifying these features is critical for understanding its function as a virulence factor and for evaluating its potential as a therapeutic target.

To address these gaps, we characterized *S. pneumoniae* HtrA using a combination of biological, biochemical, and structural approaches. Genetic knockout studies confirm that HtrA is essential for *S. pneumoniae* adhesion and virulence in infection models. Comparative proteomics between wild‐type and an HtrA knockout strain revealed numerous proteins whose levels are affected by the presence of HtrA, suggesting broad regulatory roles. Biochemically, we demonstrate that the PDZ domain is required for substrate recognition and cleavage, as shown using casein substrates—consistent with PDZ‐dependent mechanisms observed in DegP and other multimeric HtrAs. Biochemical and structural investigations reveal that *S. pneumoniae* HtrA is fully monomeric under native conditions, unlike any other HtrA family member described to date. This behavior appears to extend to other Gram‐positive homologs characterized here as well. Specifically, HtrAs from *Enterococcus faecium* (*E. faecium*) and *Staphylococcus aureus* (*S. aureus*) purified under similar conditions also behaved as monomers, suggesting that constitutive oligomerization may not be a defining feature of this subgroup. The monomeric nature of *S. pneumoniae* HtrA enabled NMR solution studies, which revealed that the PDZ domain induces a dynamic exchange between conformations—likely coupled to a substrate accessible and inaccessible state. In contrast, a catalytically inactive mutant (S234A) promoted hexamer formation, enabling cryo‐EM structural analysis and suggesting that higher‐order oligomers may arise under specific activating conditions. Together, these findings expand our understanding of Gram‐positive HtrA proteases and lay the foundation for evaluating them as potential therapeutic targets.

## 2 RESULTS

### 2.1 HtrA is essential for *S. pneumoniae* adhesion and infection

**FIGURE 2:**
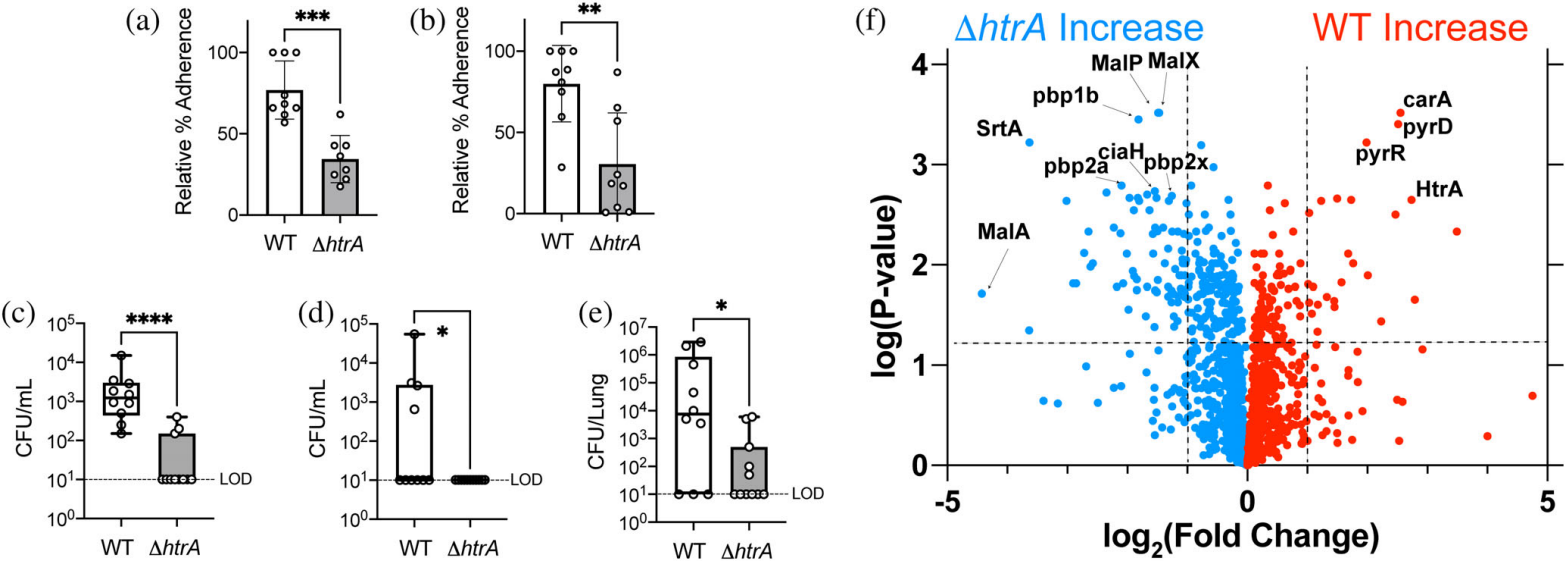
Biological consequences of *S. pneumoniae* knockout. (a) Adherence of *S. pneumoniae* WT and *DhtrA* strains to lung A549 cells. Data pooled from *n* = 3 independent experiments with nine total replicates. Error bars indicate SD. ***p < 0.001, Mann Whitney *U* Test. (b) Adherence of *S. pneumoniae* WT and *DhtrA* strains to pharyngeal D562 cells. Data pooled from *n* = 3 independent experiments with nine total replicates. Error bars indicate SD. **p < 0.001, Mann Whitney *U* Test. (c) Bacterial burden in the nasal lavage of mice infected with WT or *DhtrA* strains. Data pooled from three independent experiments with *n* = 10–11 mice/group total. Box boundaries indicate the 25th and 75th percentiles, with a horizontal line representing the median and whiskers indicating minimum and maximum values. ****p < 0.0001, Mann Whitney *U* Test. (d) Bacterial burden in the bronchoalveolar lavage (BAL) of mice infected with WT or *DhtrA* strains. *p < 0.05, Mann Whitney *U* Test. Data pooled from three independent experiments with *n* = 10–11 mice/group total. Box boundaries indicate the 25th and 75th percentiles, with a horizontal line representing the median and whiskers indicating minimum and maximum values. *p < 0.05, Mann Whitney *U* Test. (e) Bacterial burden in the lung of mice infected with WT or *DhtrA* strains. Data pooled from three independent experiments with *n* = 10–11 mice/group total. Box boundaries indicate the 25th and 75th percentiles, with a horizontal line representing the median and whiskers indicating minimum and maximum values.*p < 0.05, Mann Whitney *U* Test.

To define the biological role of HtrA in *S. pneumoniae* colonization and infection, we assessed its impact on bacterial adhesion to host cells and persistence in vivo. Compared to wild‐type (WT) *S. pneumoniae*, an *htrA* deletion strain (*ΔhtrA*) exhibited a greater than 50% reduction in adherence to pharyngeal and lung epithelial cells (Figure 2a,b), demonstrating that HtrA promotes bacterial attachment to host surfaces. Loss of HtrA also severely impaired *S. pneumoniae* persistence in a murine infection model. Following intranasal inoculation, bacterial burdens in the nasal lavage and lung tissue were diminished by orders of magnitude, while in the bronchoalveolar lavage (BAL), *ΔhtrA* burdens were completely undetectable above the limit of detection (LOD) (Figure 2c–e). The near‐complete clearance of *ΔhtrA* in the BAL highlights the essential role of HtrA in pneumococcal colonization and infection. These findings align with the work of de Stoppelaar et al. (2013), who demonstrated that HtrA is critical for *S. pneumoniae* virulence in murine infection models, while our studies further reveal that HtrA is also essential for adhesion.

### 2.2 HtrA regulates key *S. pneumoniae* proteins involved in adhesion and homeostasis

HtrA family members are multifunctional serine proteases implicated in protein quality control, including both chaperone activity and proteolysis, suggesting that these functions may be the reason its knockout in *S. pneumoniae* reduces virulence. To further delineate the molecular impact of HtrA, we performed a comparative proteomic analysis of WT *S. pneumoniae* strain D39 and an *htrA* knockout (*ΔhtrA*) strain using mass spectrometry (MS). By identifying statistically relevant differentially expressed proteins (Figure 2f), this approach provides insight into how HtrA modulates bacterial physiology and, importantly, how these regulatory mechanisms may influence adhesion and infection.

One of the most striking findings was the altered expression of penicillin‐binding proteins (PBPs), including PBP2x, which were markedly upregulated in *ΔhtrA*. This result aligns with previous studies that identified HtrA as a protease targeting PBPs for regulation (Peters et al., 2021). The downregulation of nearly all PBPs in the absence of HtrA suggests a broader role in peptidoglycan biosynthesis, which could impact bacterial cell wall integrity and resistance to β‐lactam antibiotics. Additionally, given that *S. aureus* HtrA has been proposed to chaperone PBP2a (Roch et al., 2019), it is likely that *S. pneumoniae* HtrA similarly ensures proper folding and stability of PBPs, preventing the accumulation of misfolded species directly observed here.

Sortase A (SrtA), an enzyme responsible for covalently attaching multiple adhesins to the pneumococcal surface (Jacobitz et al., 2017), was significantly upregulated in *ΔhtrA*. This result establishes a direct link between *S. pneumoniae* HtrA function and adhesion, as defects in SrtA localization or processing could contribute to the observed adhesion defects reported above in epithelial cells. Given that misfolded or improperly localized SrtA could lead to an altered distribution of surface‐exposed adhesins, this observation suggests that the impact of HtrA deletion extends beyond protein homeostasis and directly affects pneumococcal–host interactions (Pallen et al., 2001). This is reminiscent of very recent findings in *E. faecalis*, another Gram‐positive bacterium, where HtrA is required for proper pilus assembly via SrtA‐dependent processing (Colomer‐Winter et al., 2024). Thus, both our findings reported here and recent findings on multiple pathogenic Gram‐positive bacteria may indicate a broad role for their HtrAs in modulating adhesion and infection.

Beyond these likely direct effects induced within *ΔhtrA*, we observed upregulation of the two‐component regulatory system CiaR/CiaH, which controls HtrA transcription (Halfmann et al., 2007). This suggests a feedback loop, in which the absence of HtrA triggers a compensatory stress response aimed at restoring cellular homeostasis. Interestingly, the maltose metabolism operon (MalA/MalP/MalX), which is under CiaR/CiaH control, was also upregulated, reinforcing the possibility that HtrA loss induces a metabolic shift. A similar stress‐response induction was reported in *E. faecalis* following HtrA deletion (Colomer‐Winter et al., 2024), suggesting a common mechanism among Gram‐positive bacteria in which HtrA loss induces regulatory cascades to mitigate cellular stress.

Collectively, these findings establish HtrA as a key regulator of *S. pneumoniae* physiology, impacting both proteolysis‐dependent and chaperone‐mediated pathways. The observed alterations in PBPs and SrtA provide mechanistic explanations for the defects in adhesion and infection seen in *ΔhtrA* strains, while the upregulation of regulatory networks suggests broader compensatory adaptations. These include pyrimidine metabolism, including CarA, PyrR, and PyrD, that were all downregulated in *ΔhtrA*. The connection between HtrA and nucleotide metabolism remains unclear but reflects a broader role in processing key metabolic enzymes necessary for proper bacterial function beyond known targets such as the PBPs.

### 2.3 *S. pneumoniae* HtrA substrate recognition is dependent on its PDZ

**FIGURE 3:**
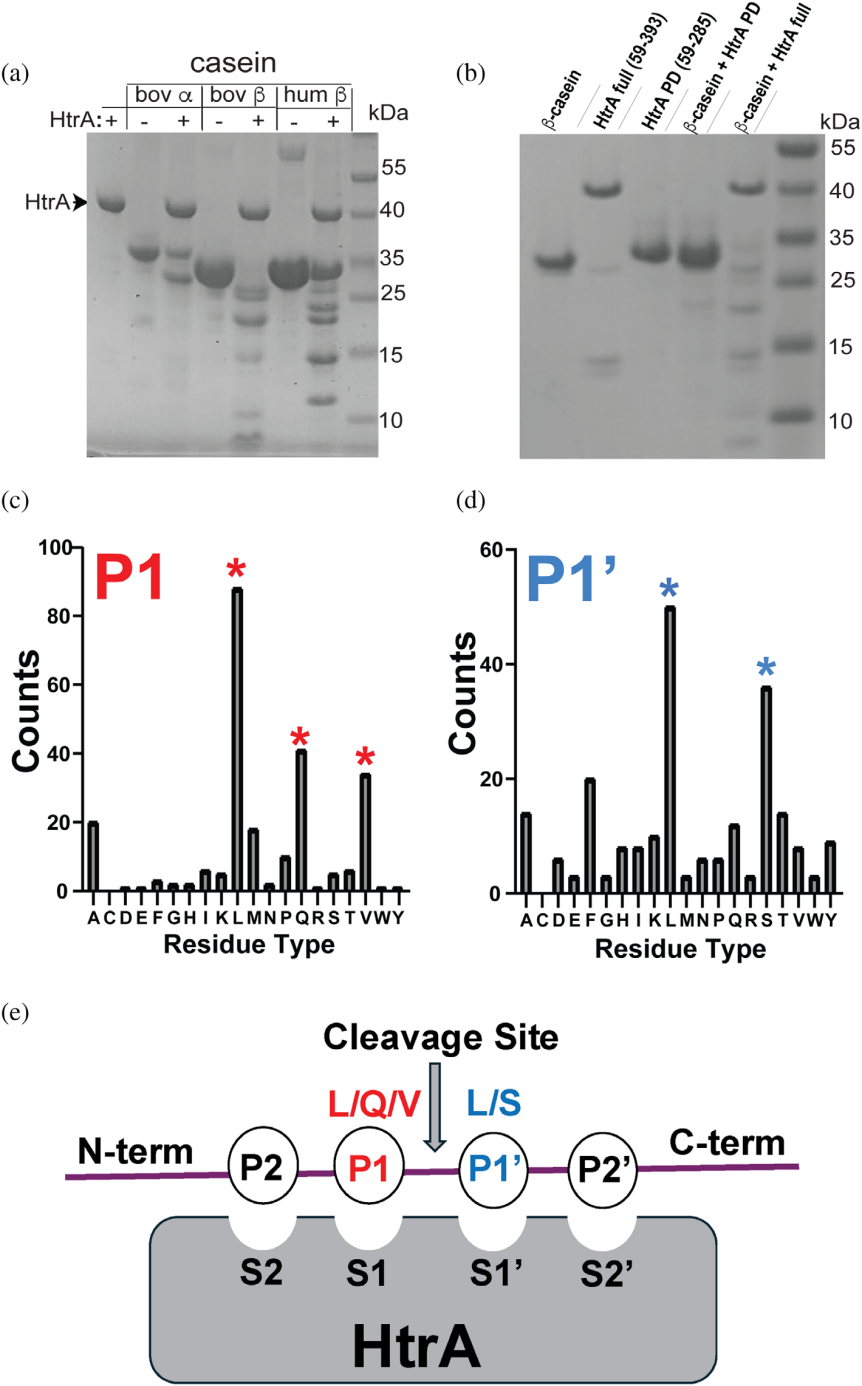
*S. pneumoniae* HtrA cleavage of caseins and preferred cleavage sites. (a) HtrA cleavage of bovine α‐casein (bov α), bovine β‐casein (bov β), and human β‐casein (hum β). (b) SDS–PAGE analysis of HtrA‐mediated β‐casein cleavage using the full HtrA ectodomain and independently produced PD and PDZ. (c) Counts derived from MS‐derived identification of HtrA cleavage peptides for P1 sites. (d) Counts derived from MS‐derived identification of HtrA cleavage peptides for P1′ sites. Concentrations of both HtrA and caseins were 10 μM with incubations at 37°C. (e) Schematic of general protease nomenclature illustrating the preferred preference for P1 and P1′ cleavage sites of *S. pneumoniae* HtrA identified here.

To investigate the catalytic properties of *S. pneumoniae* HtrA, we monitored casein cleavage that has been widely used to assess HtrA activity towards disordered proteins (Krojer et al., 2010; Zarzecka et al., 2020). Caseins are known to inhibit bacterial infection and may represent host targets that bacterial HtrAs cleave to thwart an immune response (Aniansson et al., 1990; Lönnerdal, 2004). Caseins may also serve as nutrient sources during infection (Cezairliyan & Ausubel, 2017). We employed three caseins, including commercial bovine α‐casein and β‐casein, and produced recombinant human β‐casein. All three caseins were cleaved by recombinant *S. pneumoniae* HtrA, confirming that this HtrA is catalytically active (Figure 3a). Importantly, catalytic activity was found to be dependent on the PDZ domain. Specifically, to determine the role of the *S. pneumoniae* HtrA PDZ domain, we compared the activity between the full HtrA ectodomain (residues 59–393) and the PD domain alone (residues 59–285). Robust cleavage activity of bovine β‐casein was found to be dependent on the presence of the PDZ domain (Figure 3b). These findings establish *S. pneumoniae* HtrA as a PDZ‐dependent protease, similar to the well‐characterized Gram‐negative DegP (Krojer et al., 2010), *M. tuberculosis* HtrA (Gupta et al., 2018), and *Helicobacter pylori* HtrA (Zarzecka et al., 2020). However, this contrasts with DegS, which retains protease activity independent of its PDZ domain (Wilken et al., 2004). The strict requirement for the PDZ domain in *S. pneumoniae* HtrA suggests a regulatory mechanism in which the PDZ domain modulates substrate access to the active site, potentially functioning as a gating mechanism with evidence to support this presented further below.

To further examine HtrA substrate preferences, we performed mass spectrometry (MS) analysis of the casein cleavage products. Peptides with spectral counts of 10 or more were used to determine amino acid preferences at the P1 and P1′ positions (Figure 3c,d). In serine proteases such as HtrA, catalysis is mediated by a conserved catalytic triad (His‐Asp‐Ser) that activates the serine nucleophile for peptide bond cleavage (specifically, H39‐D77‐S234 for *S. pneumoniae* HtrA). The substrate residues immediately flanking the scissile bond occupy the S1 and S1′ binding pockets of the protease active site, denoted as P1 and P1′, respectively. The side chain at the P1 position typically inserts into a hydrophobic pocket that dictates substrate specificity, while the P1′ residue lies in the adjacent pocket formed near the oxyanion hole. From our MS data for *S. pneumoniae* HtrA, the P1 position was predominantly occupied by leucine (L), glutamine (Q), or valine (V), while the P1′ position showed a strong preference for leucine (L) and serine (S) (Figure 3e). These cleavage preferences align with those reported for several other bacterial HtrAs (Gupta et al., 2018; Zarzecka et al., 2020), reinforcing the conserved nature of their substrate specificity.

### 2.4 Biochemical and biophysical studies reveal *S. pneumoniae* HtrA is monomeric

**FIGURE 4:**
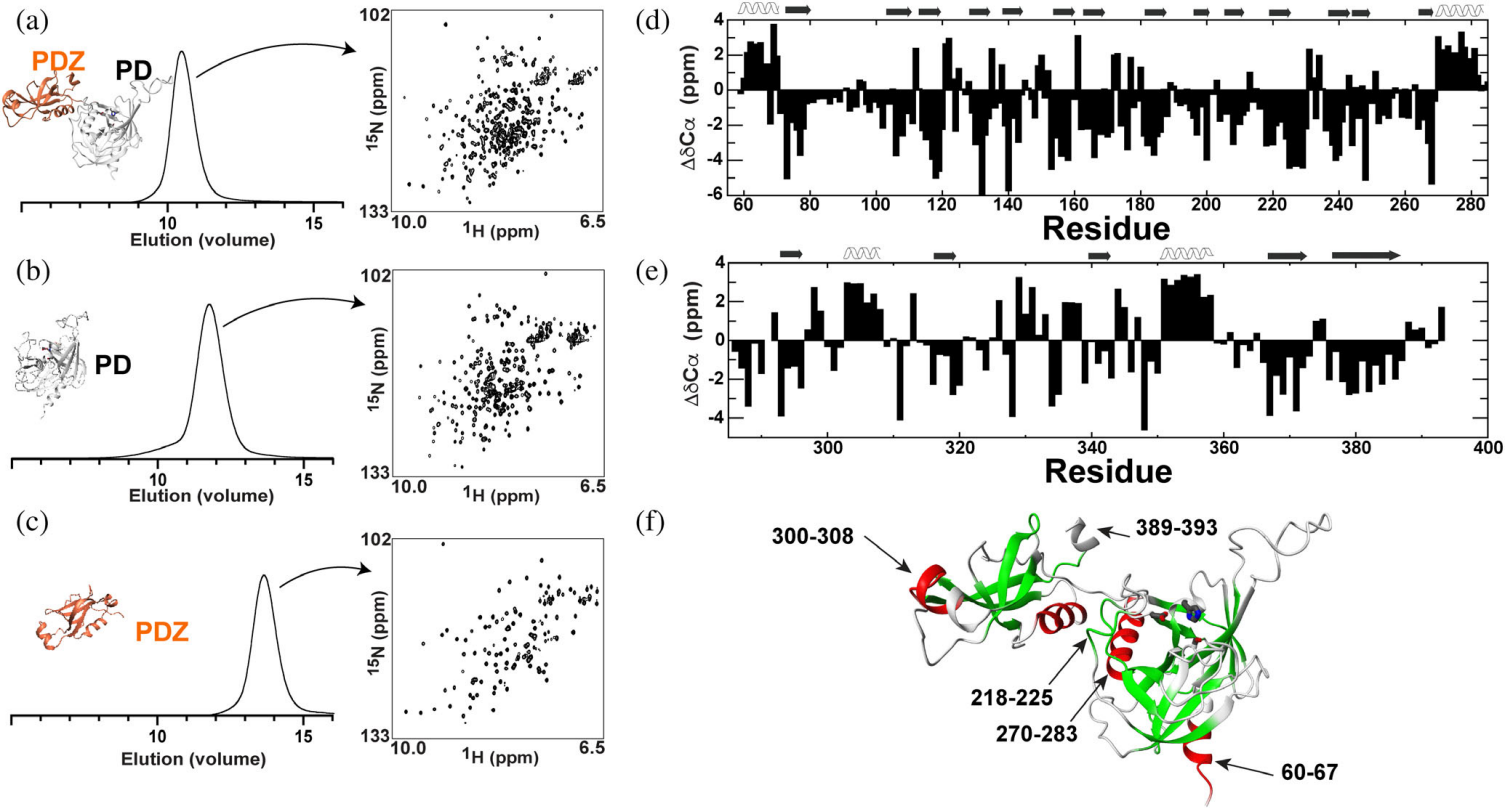
Structural studies of *S. pneumoniae* HtrA constructs. Analytical size exclusion chromatography (Superdex‐75) and ^15^N‐HSQC spectra are shown for (a) Analytical sizing (Superdex‐75) and ^15^N‐HSQC spectra (900 MHz) are shown for the full HtrA ectodomain of residues 59–393. This construct eluted at 10.4 mL that corresponds to 44 kDa estimated from a standard curve, while its real molecular weight is 36 kDa. (b) Analytical sizing (Superdex‐75) and ^15^N‐HSQC spectra (900 MHz) are shown for the HtrA PD, residues 59–285. This construct eluted at 11.8 mL that corresponds to 24 kDa estimated from a standard curve, while its real molecular weight is 23 kDa. (c) Analytical sizing (Superdex‐75) and ^15^N‐HSQC spectra (900 MHz) are shown for the HtrA PDZ, residues 285–393. This construct eluted at 13.7 mL that corresponds to 11 kDa estimated from a standard curve, while its real molecular weight is 12 kDa. (d) CA chemical shift differences to that of a random coil (Δ*δ*Cα) for the HtrA PD. (e) CA chemical shift differences to that of a random coil (Δ*δ*Cα) for the HtrA PDZ. (f) Secondary structure propensities were calculated from all backbone assignments using the Chemical Shift Index (CSI) and are delineated as β‐strand (green) or α‐helix (red). From CSI, β‐strand includes the following: 73–78, 102–110, 114–120, 130–134, 139–146, 153–158, 164–168, 180–185, 197–201, 207–210, 218–225, 238–241, 244–250, 265–269, 294–297, 318–323, 339–343, 366–373, 376–387. From CSI, α‐helix includes the following: 60–67, 270–283, 303–308, and 351–359.

As most HtrAs oligomerize in the presence of substrates, we examined the oligomeric state and structural features of *S. pneumoniae* HtrA. To this end, we compared three recombinant constructs: the full ectodomain (residues 59–393), the PD alone (residues 59–285), and the PDZ domain alone (residues 285–393). These were all selected based on the AlphaFold structure of *S. pneumoniae* HtrA. Size‐exclusion chromatography revealed that all three constructs exist as monomers and NMR spectra revealed their resonances were well‐dispersed, providing evidence for their structural integrity (Figure 4a–c). Thus, unlike most HtrAs, *S. pneumoniae* HtrA is monomeric in solution. There was also no change in the migration patterns within the pH range of 6–9, indicating that the *S. pneumoniae* HtrA remains monomeric.

We next sought to determine whether the *S. pneumoniae* HtrA NMR‐predicted secondary structure was consistent with the AlphaFold model. Comparisons of individual spectra revealed resonances within the independent domains that were similar but not identical to resonances within the full ectodomain, which is consistent with their weak interactions described within the next section. However, as spectral overlap within the full ectodomain hampered complete assignments of this 335‐residue construct (residues 59–393), we initially assigned the backbone resonances of both HtrA individual domains alone. As resonance chemical shifts are sensitive to secondary structure, especially Cα resonances, this facilitated prediction of secondary structure in solution. The near complete backbone resonances of these isolated domains were used to calculate their secondary structure propensities using the Chemical Shift Index (CSI) (Hafsa & Wishart, 2014). These CSI predictions are shown together with the CA chemical shift differences relative to a random coil (ΔδCα) (Figure 4d,e), which are largely consistent with the AlphaFold model (Figure 4f). However, there were two major differences identified between the AlphaFold and the CSI‐derived predictions. The first difference is the prediction of a helix of residues 300–308 in the AlphaFold model that CSI predicts to comprise residues 303–308. Such a subtle difference is likely reflective of the dynamic nature of this helix in solution. The second difference is residues 218–225 predicted to be somewhat helical in the AlphaFold model but a β‐strand predicted by CSI. Interestingly, our cryo‐EM studies presented for the *S. pneumoniae* HtrA S234A mutant that induces oligomeric formation are consistent with a β‐strand at this position, suggesting that this is a primary region where the AlphaFold model deviates from experimental data. However, the majority of NMR‐derived secondary structure predictions are consistent with the AlphaFold model.

We also attempted to solve the X‐ray crystal structure of HtrA but only the PDZ culminated in successful crystallization. The solved structure is highly similar to that of the NMR‐derived solution structure (Figure S1a, Table S1 with an example illustrating the high‐resolution density shown in Figure S1b) (Fan et al., 2011), other than the exact positioning of residues 300–308 and 325–338 that are likely reflective of inherently dynamic loops anchored to the rest of the protein. Both the previously solved NMR structure and our X‐ray crystal structure are identical to the AlphaFold prediction other than the specific positioning of these same regions. The overall fold of the *S. pneumoniae* HtrA PDZ domain is consistent with PDZ domains from other bacterial HtrA proteases. The *S. pneumoniae* HtrA PDZ domain adopts the circularly permuted architecture typical of HtrA family PDZs (Fan et al., 2011; Gupta et al., 2018; Krojer et al., 2010), which differs from canonical peptide‐binding PDZ domains. Like other bacterial HtrAs, the *S. pneumoniae* HtrA PDZ contains an additional C‐terminal helix that partially occludes the peptide‐binding groove, a conserved feature thought to enable regulatory flexibility rather than classical peptide recognition.

### 2.5 *S. pneumoniae* HtrA domains interact with each other

**FIGURE 5:**
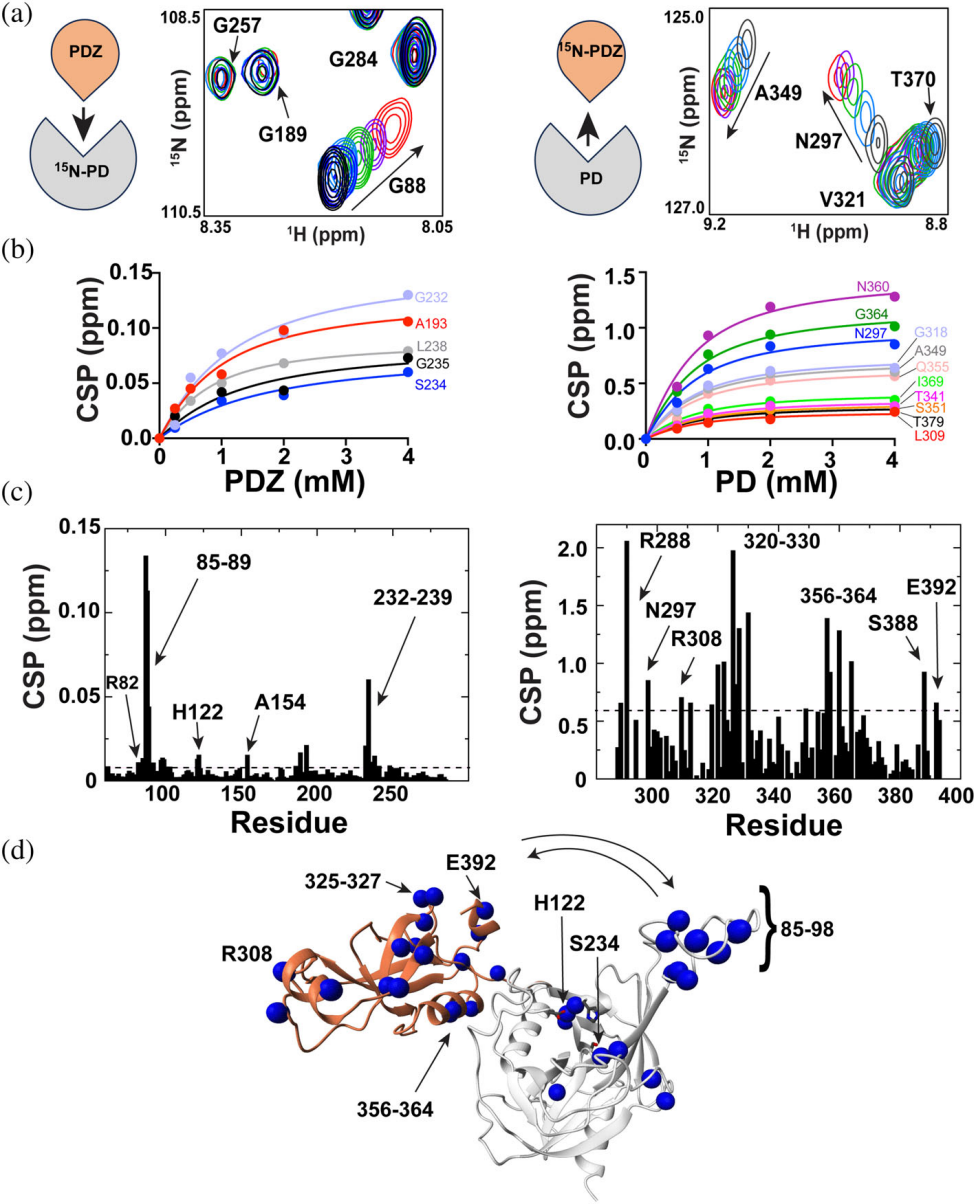
NMR titrations of the independently folded *S. pneumoniae* HtrA subdomains. (a) Selected regions illustrating titrations of ^15^N‐labeled PD titrated with unlabeled PDZ (left) and the reverse titration (right). Schematics illustrating the titration is also included. Spectra include the following: Free (black), 0.25 mM (blue, only in PD titration with PDZ), 0.5 mM (sky blue), 1 mM (green), 2 mM (mauve), and 4 mM (red). (b) Binding isotherms for each titration were used to quantify the binding affinities from multiple resonance CSPs. From global fits for each titration, a *K*_D_ of 480 ± 43 μM was derived (left) and a 1.0 ± 0.5 mM was derived (right). (c) CSPs plotted for each titration between no titrant and 4 mM titrant for the PD (left) and the PDZ (right). For the PD domain (left), the average CSP was 0.006 ppm and standard deviation 0.013 ppm. This average plus a half standard deviation of 0.013 ppm is delineated (dashed line). For the PDZ domain (right), the average CSP was 0.44 ppm and standard deviation 0.41 ppm. The average plus a half standard deviation is of 0.63 ppm is delineated (dashed line). (d) From titrations of the independent domains, CSPs larger than the average plus one standard deviation are mapped onto the HtrA model (blue spheres). All NMR data were collected at 900 MHz at 35°C using 500 μM labeled domains.

To determine whether the PD and PDZ domains interact, we performed NMR titrations with the separately purified domains. Titrations with each ^15^N‐labeled domain and the other unlabeled domain identified their site‐specific interactions (Figures 5a and S1c,d). The chemical shift perturbations (CSPs) from each of these titrations were used to fit their respective binding isotherms, revealing dissociation constants (*K*_D_) of these separate individual domains within the mid‐micromolar (μM) range at 35°C (Figure 5b). Specifically, 480 ± 43 μM was derived from a global fit of the PD titrated with the PDZ, while 1.0 ± 0.5 mM was derived from the PDZ titration with the PD. CSP mapping identified key regions that likely comprise the interaction surface, including residues 78–100 (also referred to as LoopA) and the catalytic triad residues H122, D152, and S234 (Figure 5c,d). These data indicate that the PDZ domain directly engages the PD in the absence of a substrate, potentially regulating protease activity.

Considering the relatively small amide CSPs incurred for the PD domain upon PDZ binding, compared to the reverse binding that induces larger amide PDZ CSPs, we further probed these domain interactions utilizing NMR‐based methods. The underlying basis for these differences is likely due to the malleable nature of PDZ domains in general that facilitate large‐scale conformational changes upon binding (Münz et al., 2012). This suggests that the PDZ undergoes larger conformational changes than more subtle and localized changes for the PD domain. We therefore sought to measure the Cα CSPs that would be sensitive to secondary structural changes between PD/PDZ interactions that would sample both a “closed” form and an “open” form (visualized in the predicted AlphaFold structure). To this end, we attempted to assign the backbone resonances of the entire ectodomain of HtrA residues 59–393. This provided Cα differences between the PD alone and in the context of the full ectodomain (Figure S1e). Similar efforts to identify changes of the PDZ were hampered by a lack of several residue assignments in the context of the full ectodomain and thus, we compared the PDZ alone and in the context of a biochemically saturated complex with the PD (Figure S1f). As predicted, large Cα CSPs were identified for the PDZ domain, indicative of larger conformational changes. Collectively, the Cα CSPs for both the PD and PDZ comparisons map to similar regions of the amide CSPs (Figure S1g), providing further evidence for conformational changes between open and closed conformations. Finally, the amides between the individual domains alone and in the context of the full HtrA ectodomain of residues 59–393 were also quantified (Figure S1h), which identified many CSPs within the PD active site (Figure S1i). These results provide additional evidence that PD–PDZ interactions in the full ectodomain induce conformational changes across the protease domain. Further attempts to identify intermolecular information, such as filtered‐edited NOEs, were unsuccessful, potentially owing to the relatively weak interaction between these two domains.

The fact that the *S. pneumoniae* HtrA PDZ is required for activity while simultaneously binding to regions of the PD that could occlude the active site suggests that these domains may be in a dynamic equilibrium between accessible and non‐accessible forms. While *M. tuberculosis* HtrA is a trimer, it has been crystallized in both open and closed forms (Gupta et al., 2018). Considering that *S. pneumoniae* HtrA is a monomer in solution, this facilitates directed NMR relaxation experiments aimed at elucidating the dynamic nature of these interactions in what follows.

### 2.6 *S. pneumoniae* HtrA undergoes a dynamic exchange induced by its PDZ

To investigate the conformational dynamics of *S. pneumoniae* HtrA, we utilized NMR‐based methods, including ^15^N‐HSQC spectral comparisons and R_2_‐CPMG relaxation dispersion experiments, to probe the exchange between its open and closed states. These experiments provide direct evidence that HtrA undergoes micro‐millisecond (μs–ms) motions that facilitate PD and PDZ domain interactions.

**FIGURE 6:**
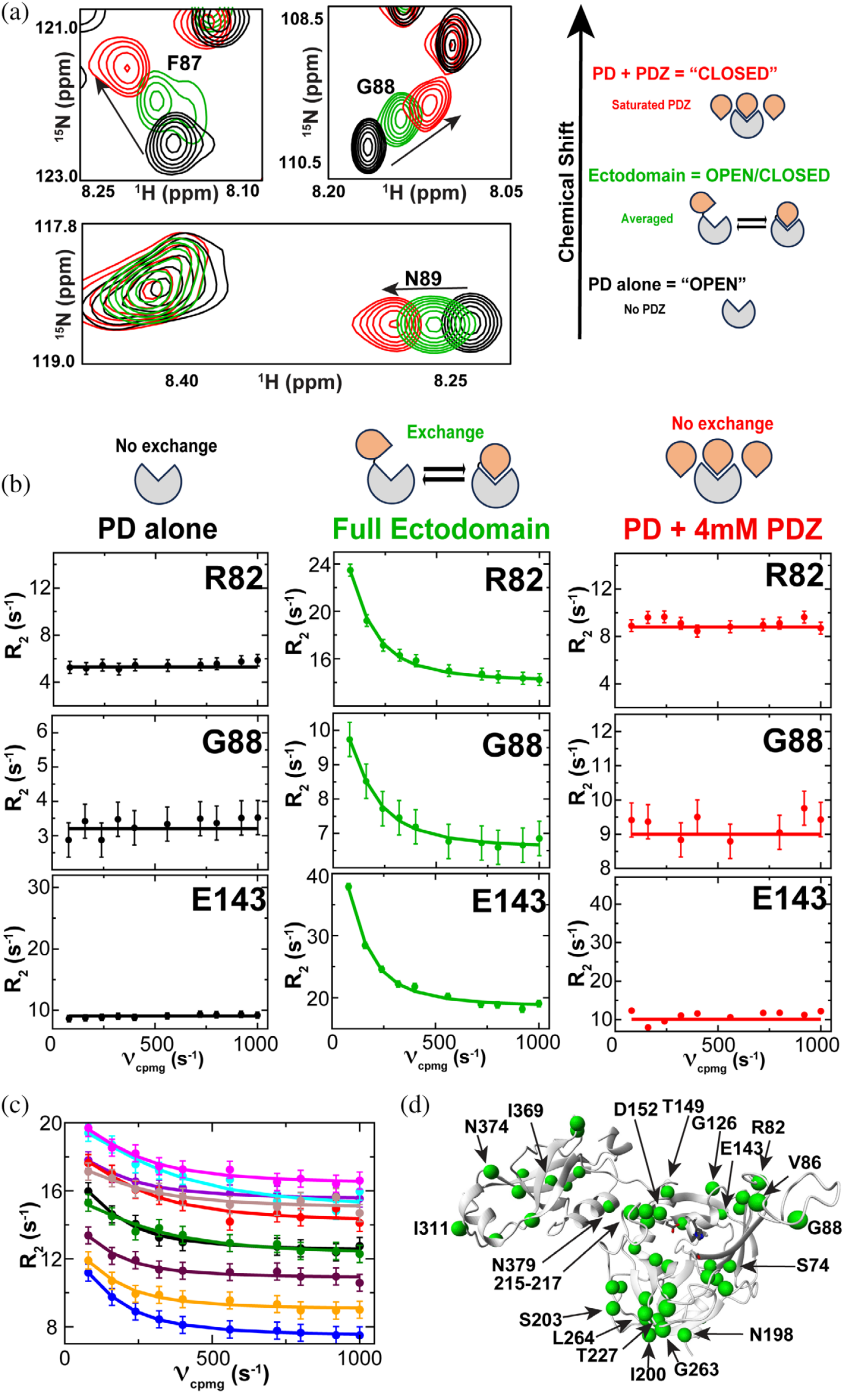
Spectral comparisons and relaxation experiments of *S. pneumoniae* HtrA. (a) Examples of systematic shifts for LoopA residues within the free PD (black), the full ectodomain (green), and the biochemically saturated PD with 4 mM PDZ (red) for F87, G88, and N89. A schematic illustrating the potential model of HtrA existing as “opened” and “closed” is illustrated based on the observation that resonances of the full ectodomain fall in between. (b) R_2_‐CPMG dispersions of the PD alone, the full ectodomain, and the biochemically saturated PD with a cartoon drawing above describing the observed exchange. (c) R_2_‐CPMG dispersions of the full ectodomain. Shown are global fits for S74 (blue), V86 (blue), G126 (violet), D152 (cyan), G215 (maroon), T227 (red), I311 (brown), N360 (magenta), I369 (orange), and N379 (green). Further R_2_‐dispersions are also provided (Figure S2). (d) Resonances are mapped onto the predicted HtrA structure that exhibit R_2_‐CPMG dispersion (green spheres), noting that backbone resonances still await completion for the full HtrA ectodomain. All data were collected at 900 MHz at 35°C.

HSQC spectral comparisons revealed that resonances within LoopA exhibit chemical shifts within the full ectodomain that are in between their resonance. Positions in the independently folded PD and those observed under saturating conditions, where the PD is bound to an excess of the PDZ domain (Figure 6a, left). This pattern is consistent with a largely two‐state conformational exchange in which the full HtrA ectodomain exists in a dynamic equilibrium that samples a dominant two‐state equilibrium. The fact that these LoopA resonances of the full HtrA ectodomain fall roughly in between the extreme states of the PD alone (i.e., potentially opened) and the saturated PD (i.e., potentially closed) suggests that the PDZ is sampling two conformations on the NMR timescale, resulting in averaged chemical shifts that reflect rapid interconversion. This intermediate sampling of chemical shifts is schematically depicted as the sampling of opened and closed conformations (Figure 6a, right), but the exact estimate of the populations is unknown. While we note that this phenomenon is not observed for other resonances within the PD, most other resonances exhibit very small CSPs with the addition of the PDZ and could also be impacted by allostery that we experimentally show occurs below.

As a complimentary means to quantify the underlying dynamic that may lead to this exchange, we performed R_2_‐CPMG dispersion experiments, which provide insight into μs–ms timescale dynamics. No exchange was detected for residues within the PD alone (Figure 6b, left) or in the PD saturated with the PDZ domain (Figure 6b, right), indicating that in these conditions, HtrA exists predominantly in one conformation, that is, open or closed, respectively. However, in the context of the full HtrA ectodomain, significant R_2_‐CPMG dispersion was observed, confirming that residues affected by the PD–PDZ interaction undergo conformational exchange in the μs–ms timescale (Figure 6b, middle). This exchange between conformations induced measurable R_2_‐CPMG dispersion for residues within both the PDZ and the PD (Figure 6c). Many of these residues are predicted to be directly at the PD–PDZ interface, such as LoopA residues R82, V86, and G88. These residues exhibit CSPs for domain titrations (Figure 5c) and comparisons between the isolated domains and full ectodomain (Figure S1i). Additionally, many of the residues that exhibit exchange are much more distant from the interaction surface, which includes residues on the opposite face of the PD, such as I200, S203, G263, and L264. Thus, such relaxation experiments highlight the broader allosteric coupling of this exchange process. Most R_2_‐CPMG dispersions could be globally fit with an extracted 950 ppm ± 200 s^−1^, with a relatively large range of the “minor” population estimated at 30%–45% (Figure S2). However, as we and others have shown before (Lee et al., 2023; Paukovich et al., 2018), such dynamics are likely only partially coupled, referred to as “segmental” in nature (McDonald et al., 2012), and may not explicitly reflect a single dynamic process.

These results demonstrate that *S. pneumoniae* HtrA undergoes conformational exchange on the μs–ms timescale, involving the PD and PDZ domains. Specifically, while the HSQC spectral comparisons of LoopA and nearby residues indicate an averaging between conformations (Figure 6a), the R_2_‐CPMG‐derived chemical shift differences from the fits do not match those observed in the domain titrations. Such differences between the extracted chemical shift differences from R_2_‐CPMG dispersions and the measured CSPs could reflect complications due to allostery or other phenomena. For example, the conformational exchange observed in the full‐length ectodomain may not necessarily represent a simple two‐state equilibrium between fully open and fully closed conformations. Instead, the dynamics could reflect transitions involving partially populated or alternative intermediate states, distinct from the isolated PD and PDZ complexes, which could also underlie the larger conformational sampling of open and closed conformations. These findings underscore the complexity of PD/PDZ interactions in *S. pneumoniae* HtrA and suggest that the enzyme samples a broader conformational ensemble, potentially relevant for its regulatory function.

### 2.7 The active‐site *S. pneumoniae* HtrA S234A mutation is globally coupled

Allosteric coupling is a hallmark of many HtrA family members, including *E. coli* DegS and *M. tuberculosis* PepD, where long‐range PDZ‐to‐active‐site coupling is proposed to promote proteolytic activation (Gupta et al., 2023; Wilken et al., 2004). To determine whether such allosteric communication exists in *S. pneumoniae* HtrA, we mutated the catalytic serine to alanine (S234A) in both the isolated PD and the full ectodomain.

**FIGURE 7:**
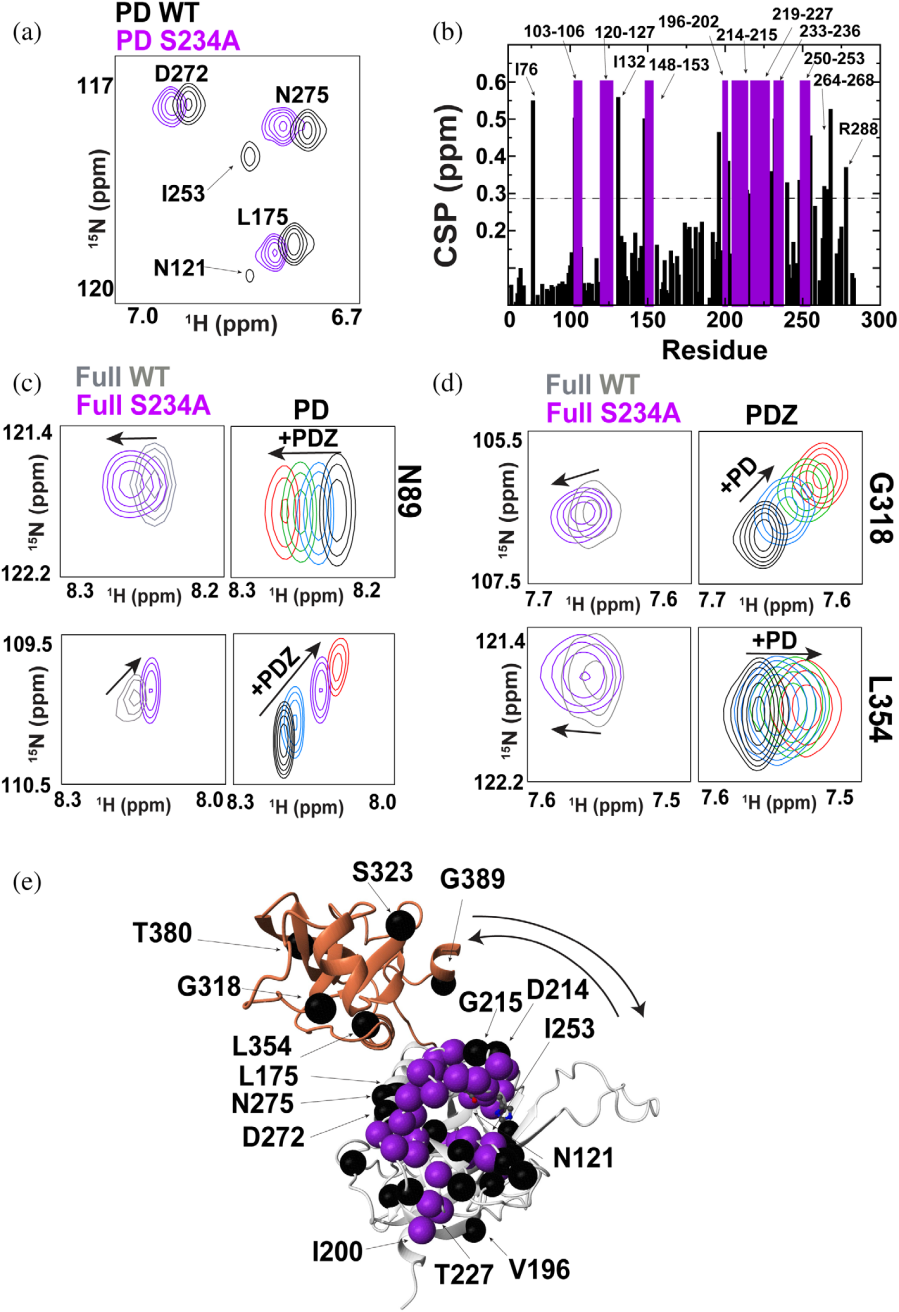
Global effects induced by the *S. pneumoniae* HtrA S234A mutant monitored via NMR. (a) Selected region of ^15^N‐HSQC for PD WT (black) and PD S234A (purple), illustrating both shifts and the disappearance of resonances. (b) Global amide CSPs between PD WT and PD S234A (black) with resonances that either disappear or exhibit CSPs too large to identify (purple). (c) Examples of full HtrA S234A ectodomain induced CSPs (purple) relative to the full HtrA WT ectodomain (gray) that are in the same direction of the titrations of the PD (black) with 0.5 mM PDZ (blue), 1 mM PDZ (green), and 4 mM PDZ (red). (d) Examples of full HtrA S234A ectodomain induced CSPs (purple) relative to the full HtrA WT ectodomain (gray) that are in the opposite direction of the titrations of the PDZ (black) with 0.5 mM PD (blue), 1 mM PD (green), and 4 mM PD (red). (e) Amide CSPs between full HtrA WT ectodomain and the S234A mutation indicating measured CSPs (black spheres) and those that shift to unidentified positions (purple spheres) mapped onto the PD. Additional resonances within the PDZ domain in the context of the full HtrA ectodomain that shift with the S234A mutation are also shown (black spheres). The average CSP was 0.15 ppm and standard deviation was 0.14 ppm (dashed line indicates the sum).

Although most of the HtrA S234A mutant forms a hexamer in the full ectodomain (described in the next section), all NMR analyses were performed using the monomeric fraction purified by size‐exclusion chromatography. Because the monomeric and hexameric species are readily separable (Figure 7a), their interconversion occurs on a timescale much slower than that of NMR chemical exchange. The observed residue‐specific CSPs are therefore unlikely to arise from monomer‐hexamer exchange and instead reflect intrinsic effects induced by the S234A mutation. Therefore, NMR was used to map the global changes induced by the S234A mutation for both the PD that remains monomeric and the full ectodomain monomer.

Within the PD alone, chemical shift perturbations (CSPs) relative to WT were widespread (Figure 7b), and several resonances were lost entirely, consistent with increased dynamic exchange in the μs–ms regime. These findings suggest that the mutation enhances local flexibility in the PD, particularly in residues already broadened in the WT enzyme.

When extended to the full ectodomain, the S234A mutation induced CSPs in both the PD and PDZ domains. Notably, the PD shifts followed the same trend as seen when titrating PDZ into the PD, consistent with a more PDZ‐engaged, or closed, conformation (Figure 7c). In contrast, the PDZ domain exhibited CSPs in the opposite direction of those observed during PDZ titration with PD (Figure 7d), suggesting that the PDZ adopts a more disengaged or open state. This unexpected asymmetry suggests that the S234A mutation perturbs the internal coupling between domains rather than stabilizing a single defined conformation. Instead, the mutation may favor a misaligned or intermediate ensemble that alters domain–domain interactions and potentially primes the protein for higher‐order assembly, as observed by cryo‐EM (described below). However, it should be noted that severe overlap within the full ectodomain precludes a more expansive quantitative study and any conclusions should be made with caution.

To illustrate this distributed allosteric response, S234A‐induced CSPs for the isolated PD are mapped onto the structural model of *S. pneumoniae* HtrA together with the subset of PDZ CSPs that could be extracted from the comparisons of the full ectodomain (Figure 7e).

### 2.8 The active site *S. pneumoniae* HtrA S234A mutation induces hexameric formation

**FIGURE 8:**
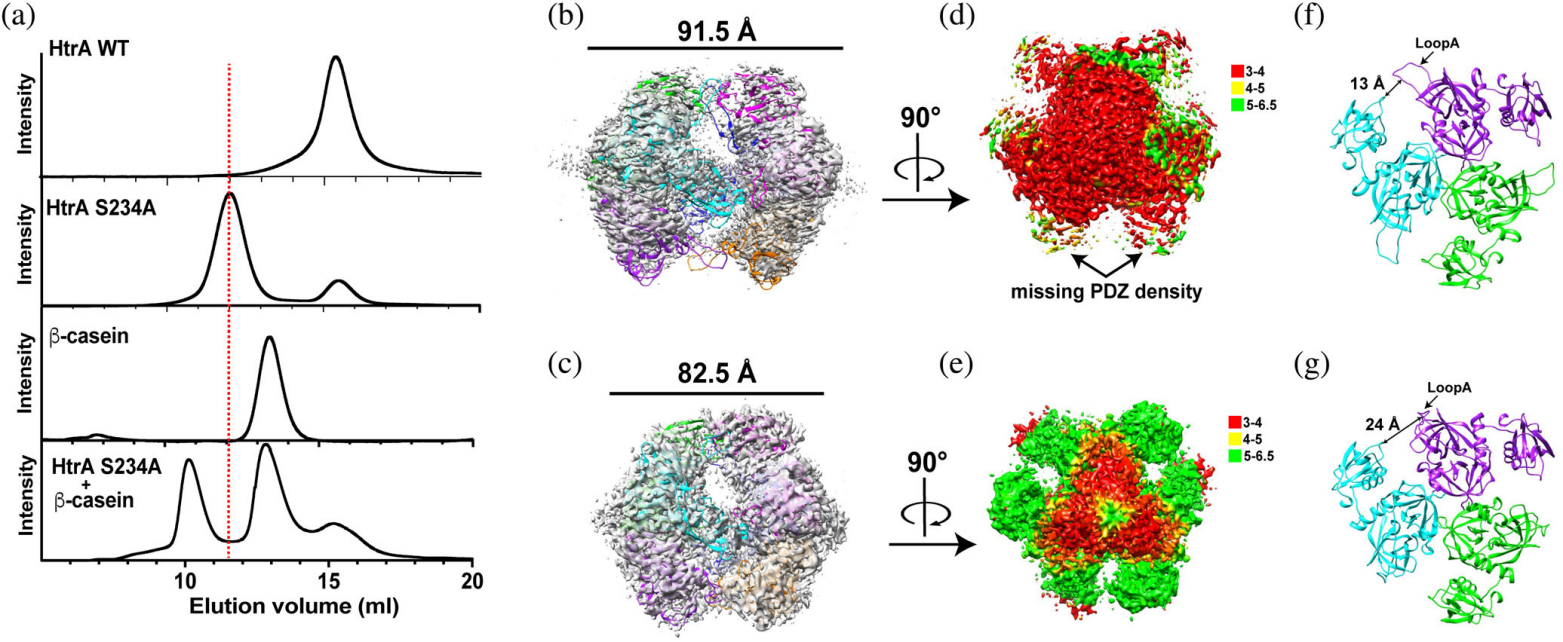
Cryo‐EM analysis of the *S. pneumoniae* HtrA S234A mutant‐induced oligomer. (a) Size‐exclusion chromatography of HtrA WT, HtrA S234A, β‐casein, and a mixture of HtrA S234A with excess β‐casein. (b) Cryo‐EM density map and corresponding atomic model of substrate‐free HtrA S234A with the side view of trimer/trimer interaction. (c) Cryo‐EM density map and corresponding atomic model of the HtrA S234A/β‐casein complex with the side view of trimer/trimer interaction. (d) Local resolution of apo *S. pneumoniae* HtrA S234A colored at 3–4 Å (red), 4–5 Å (yellow), and 5–6.5 Å (green). (e) Local resolution of the HtrA S234A/β‐casein complex colored at 3–4 Å (red), 4–5 Å (yellow), and 5–6.5 Å (green). (f) Model of the apo *S. pneumoniae* HtrA S234A trimer in the same orientation as (d). (g) Model of the HtrA S234A/β‐casein complex trimer in the same orientation as (e).

In addition to the long‐range allosteric impact induced by the HtrA S234A mutation in the monomer, there was a significant population that also shifts to a larger oligomeric form found upon purification (Figure 8a). The migration pattern of this oligomer suggests it is a hexamer that is consistent with hexamer formation observed for some Gram‐negative HtrAs (Song et al., 2022). However, unlike these Gram‐negative HtrAs that form hexamers with two PDZ modules, such as *E. coli* DegP and DegQ, HtrAs with a single PDZ normally do not form hexamers. For example, HtrA hexamer formation with a single PDZ has only been observed in the Gram‐negative *Synechocystis* sp. HhoA (Hall et al., 2017). HtrAs with a single PDZ, such as *E. coli* DegS and *M. tuberculosis* HtrA, both form trimers (Gupta et al., 2018; Wilken et al., 2004). Thus, the observation of the hexamer in *S. pneumoniae* HtrA is relatively rare.

We used cryo‐EM to directly visualize the *S. pneumoniae* HtrA S234A hexamer. As the HtrA S234A mutation results in an inactivated serine protease, we also incubated this mutation with bovine β‐casein, which resulted in a migratory shift indicative of complex formation (Figure 8a). Hexameric formation for both the substrate‐free HtrA S234A and the complex with β‐casein could be observed by single‐particle reconstructions (Figures S3 and S4). In our cryo‐EM reconstructions of hexameric HtrA S234A (Figure 8b,c), we observed poorly defined density for the PDZ domains, consistent with significant conformational flexibility that could be identified by their local resolution maps (Figure 8d,e). Each of the two trimers comprising three PDs could be accurately fit (Figure S5a,b). In contrast, densities were too poor to accurately model the PDZ domains and thus, the X‐ray crystal structure that we determined here was fit within the cryo‐EM densities. These findings also align with previous structural studies of bacterial HtrA proteases that identify PD interactions forming a trimer with PDZ domains exhibiting conformational heterogeneity (Figure S5a,b). For example, the crystal structure of hexameric DegP revealed that, while the protease domains were also well ordered, its two PDZ domains were only partially resolved, consistent with high mobility in the hexamer (Krojer et al., 2002). In contrast, cryo‐EM and crystallographic studies of larger DegP and DegQ assemblies, such as 12‐ and 24‐mers, showed their two PDZ domains are further stabilized by inter–subunit interactions (Krojer, Sawa, et al., 2008; Malet et al., 2012). These observations support a model in which PDZ domain flexibility is an inherent feature, especially in smaller hexamer assemblies, but can become structurally ordered in only some cases of substrate binding and oligomer activation.

Several overall conclusions can be made from our cryo‐EM studies of hexameric HtrA S234A. First, analogous to other HtrA/substrate bound interactions, the substrate is not observed within the *S. pneumoniae* HtrA S234A complex, likely owing to conformational flexibility that may also include multiple binding sites (Krojer, Sawa, et al., 2008; Malet et al., 2012). Second, although density is too weak to model the PDZ accurately within the cryo‐EM derived model, the density of the PDZ visibly changes its orientation in the bound state with a concomitant change in the orientation of the PD LoopA region (Figure 8f,g). Third, a comparison of both substrate‐free and β‐casein bound *S. pneumoniae* HtrA S234A structures reveals that the two trimers come within closer proximity with the substrate, compressing the diameter of the hexamer from 91 to 81 Å (Figure S5c,d). Thus, there is a substrate‐dependent contraction of the hexamer. Fourth, despite poor densities for the PDZ domains, the trimer appears to be formed strictly through PD interactions. This is similar to DegS that also does not comprise PDZ stabilizing interactions of its trimer (Figure S5e), yet distinct from others like *M. turberculosis* HtrA that do employ PD/PDZ interactions within the trimer (Figure S5f). Fifth, and as opposed to the lack of PD/PDZ interactions within the trimer, the *S. pneumoniae* HtrA S234A trimer–trimer interactions are stabilized by PD/PDZ interactions. Specifically, the PD LoopA interacts with the PDZ helix of residues 300–308 within the other trimer, although the explicit structure is too weak to model without higher resolution that would necessitate mutagenesis to further define (Figure S5c,d). However, this is the same location of the PDZ helix that is in different positions between the previous NMR structure and our X‐ray crystal structure (Figure S1a), consistent with its dynamic positioning due to its flexible tethering within the PDZ. Thus, the HtrA S234A mutation induces a significant fraction of the full ectodomain to form a hexamer that is likely stabilized by a unique PD/PDZ interaction not yet observed within the wider HtrA family.

### 2.9 Interrogating *S. pneumoniae* HtrA interactions

**FIGURE 9:**
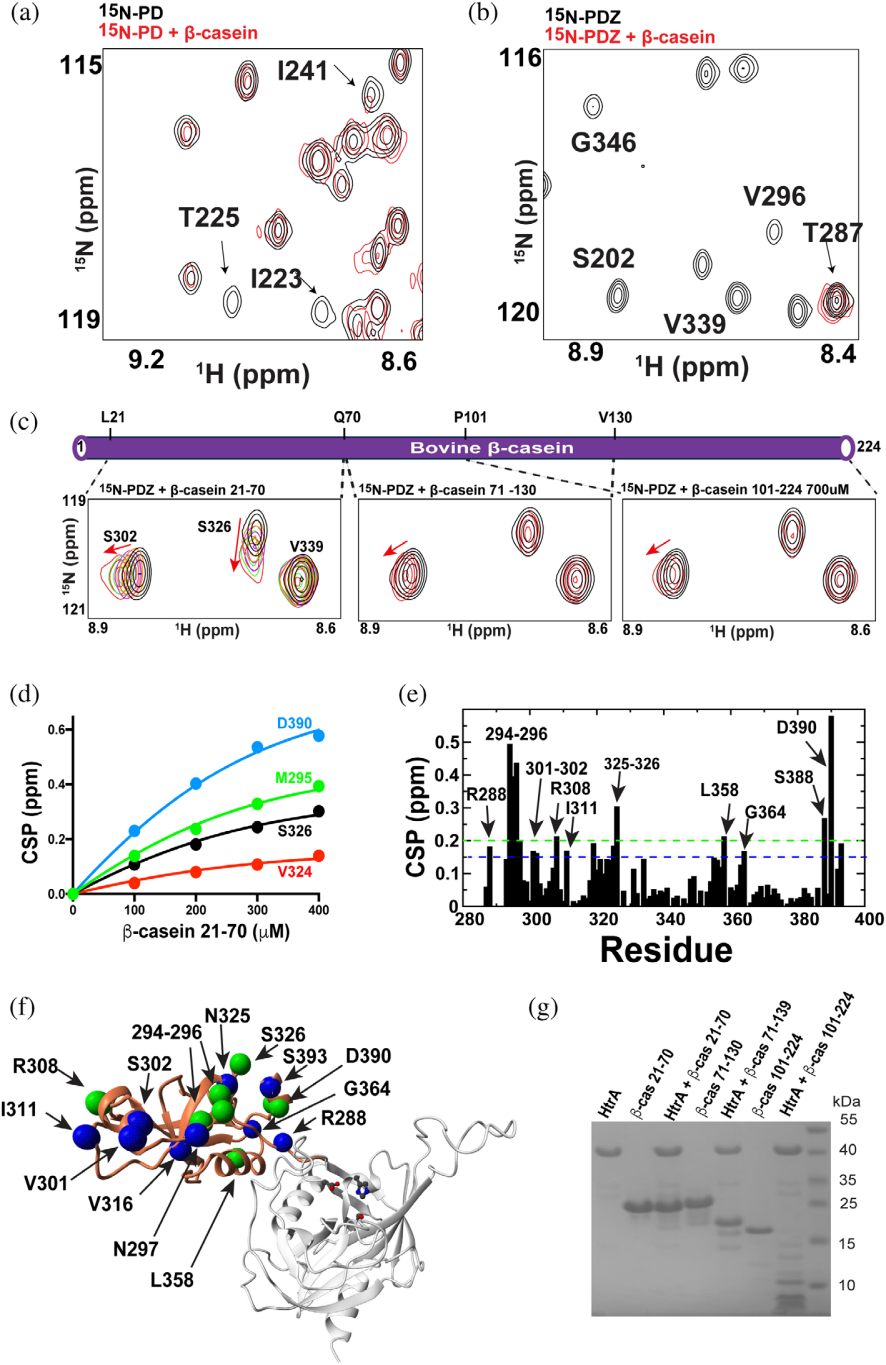
*S. pneumoniae* HtrA domain‐specific interactions with β‐casein. (a) Selected region of ^15^N‐HSQC for 250 μM HtrA PD alone (black) and in the presence of 500 μM full‐length β‐casein (red). (b) Selected region of ^15^N‐HSQC for 250 μM HtrA PDZ alone (black) and in the presence of 500 μ full‐length β‐casein (red). (c) Selected region of ^15^N‐HSQC for a titration of 250 μM HtrA PDZ with recombinantly purified regions of β‐casein that include residues 21–70 (left), residues 71–130 (middle), and residues 101–224 (right). Intermediate titration points are only shown for β‐casein residues 21–70, while only the free and final 400 mM concentrations of added β‐casein fragments are shown for the others. (d) Binding isotherms of ^15^N‐PDZ from titrations with β‐casein residues 21–70 are shown that were globally fit with an extracted *K*_D_ of 215 ± 35 μM. Only a subset of the nearly two dozen amide shifts are shown for clarity. (e) Amide CSPs between ^15^N‐PDZ alone and in the context of 400 μM β‐casein residues 21–70. The average CSP was 0.10 ppm with a standard deviation of 0.10 ppm. Dashed lines correspond to the average plus one standard deviation (0.20 ppm, green) and ½ standard deviation (0.15 ppm, blue). (f) CSPs are mapped onto the HtrA PDZ within the model structure with spheres color‐coded and described in (e). (g) SDS–PAGE analysis of full HtrA ectodomain and each of the three β‐casein constructs are shown both alone and incubated together. β‐Casein residues 21–70 and residues 71–130 also comprise the hSUMO tag for visualization on the gel and β‐casein residues 101–224 comprises an N‐terminal 6xHis tag.

We further interrogated the WT *S. pneumoniae* HtrA interactions through NMR using bovine β‐casein. We focused on the separately purified HtrA domains that have good dispersion and do not suffer from the resonance overlap issues that occur within the full HtrA ectodomain. Using ^15^N‐labeled PD and PDZ, the addition of bovine β‐casein culminated in resonance‐specific line‐broadening for the PD (Figure 9a), in contrast to nearly the complete loss of resonances for the PDZ (Figure 9b). These results suggest that the partial loss of resonance intensities for the PD domain may be due to very weak interactions that slow tumbling (and lead to a loss of signal). In contrast, the near complete disappearance of the PDZ resonances may be due to its engagement of multiple β‐casein sites, which we confirm occurs below, while a further loss of signal may be due to slowed tumbling due to binding. These results are also consistent with our gel‐based findings presented above whereby the PDZ domain is largely responsible for substrate recognition.

To define the regions of β‐casein involved in binding, we tested three recombinant β‐casein fragments (residues 21–70, 71–130, and 101–224). The N‐terminal segment (21–70) induced the largest CSPs in the PDZ domain (Figure 9c–e) with mid–micromolar affinity (Figure 9d). Interestingly, this fragment was not cleaved by full‐length HtrA, whereas the weaker‐binding fragments were (Figure 9g), suggesting that strong PDZ engagement alone is not sufficient for catalysis. This likely reflects non‐productive binding, in which the substrate interacts with the PDZ in an orientation that does not position the scissile bond near the protease active site. Alternatively, such contacts may represent stabilizing or adhesion‐like interactions that support substrate recognition rather than cleavage.

Finally, we tested the binding of a short hydrophobic peptide similar to that previously used in titrations to the HtrA PDZ (Fan et al., 2011). This peptide, suc‐AFPF‐pNA, induced only minor CSPs to the HtrA PD. In contrast, suc‐AFPF‐pNA induced much larger CSPs to the PDZ domain (Figure S6b), which could be used to quantify its moderate affinity to the PDZ (Figure S6c) and show its interact was a distinct surface compared to β‐casein (Figure S6d,e). In contrast, no binding was detected for folded proteins such as PBP2x or lysozyme (Figure S6g), supporting a preference for unfolded substrates. Collectively, these results reinforce that substrate engagement by *S. pneumoniae* HtrA occurs primarily through its PDZ domain, which selectively recognizes unfolded polypeptides via multiple low‐affinity interactions.

### 2.10 Other Gram‐positive HtrAs are monomeric in solution

Despite extensive structural studies of Gram‐negative HtrAs, little is known about the quaternary structures of their Gram‐positive counterparts. In fact, to our knowledge, no prior biochemical studies have examined the oligomeric states of purified HtrAs from *E. faecium* or *S. aureus*. To address this gap and determine whether monomeric behavior observed for *S. pneumoniae* HtrA is a broader feature of Gram‐positive bacteria, we cloned, expressed, and purified the recombinant ectodomain HtrA homologs from *E. faecium* and *S. aureus*, which share 56% and 35% identity to *S. pneumoniae* HtrA, respectively. Analytical size‐exclusion chromatography revealed that, like *S. pneumoniae* HtrA (Figure S7a), the other Gram‐positive HtrAs eluted as monomers under native conditions (Figure S7b,c). While higher‐order oligomerization could occur under alternative conditions or at elevated concentrations, these results suggest that monomeric architecture may be a conserved property among Gram‐positive HtrAs. These results also further emphasize a structural divergence from the well‐characterized trimeric and multimeric forms observed in many Gram‐negative HtrAs.

## 3 DISCUSSION

### 3.1 HtrA functions as a quality control protease in *S. pneumoniae*

Our proteomic and biochemical data collectively suggest that *S. pneumoniae* HtrA plays a central role in maintaining intracellular protein homeostasis, particularly by targeting misfolded or unfolded proteins for degradation. The upregulation of stress‐related proteins and chaperone systems in the *ΔhtrA* strain, along with the observed destabilization of key folding‐dependent proteins such as PBPs and SrtA, supports a model in which HtrA functions as a quality control protease that preserves the integrity of both cytoplasmic and membrane‐associated proteins. Similar roles in protein quality control have been ascribed for the wider HtrA family, which includes human HtrAs (Zurawa‐Janicka et al., 2017) and Gram‐negative HtrAs (Backert et al., 2018). Even more relevant, our proteomic data confirm that the unfolded targets overlap with those of other Gram‐positive HtrA family members, such as *S. aureus* HtrA1, which targets PBPs (Roch et al., 2019), and *E. faecium* HtrA, which targets SrtA (Colomer‐Winter et al., 2024). Furthermore, the strict requirement of the PDZ domain for activity and its preferential binding to hydrophobic, unstructured substrates, exemplified here by β‐casein peptide engagement, highlights a mode of substrate recognition tailored to unfolded conformations. While our data primarily support a role in intracellular protein surveillance, it is also possible that HtrA engages additional extracellular or host‐associated substrates involved in adhesion or virulence.

### 3.2 *S. pneumoniae* HtrA is an inherently dynamic monomer

Unlike the vast majority of HtrA family members, which form stable trimers and higher‐order oligomers based on this trimer unit, *S. pneumoniae* HtrA was shown here to behave as a monomer in solution. This monomeric state is shared by other Gram‐positive homologs that we purified here, including those from *E. faecium* and *S. aureus*, suggesting that constitutive oligomerization is not a defining feature of this subgroup. Despite its monomeric nature, *S. pneumoniae* HtrA exhibits substantial conformational flexibility, consistent with an allosterically regulated system in which much of the protein appears coupled to the active site, as observed by NMR studies of the S234A mutant.

**FIGURE 10:**
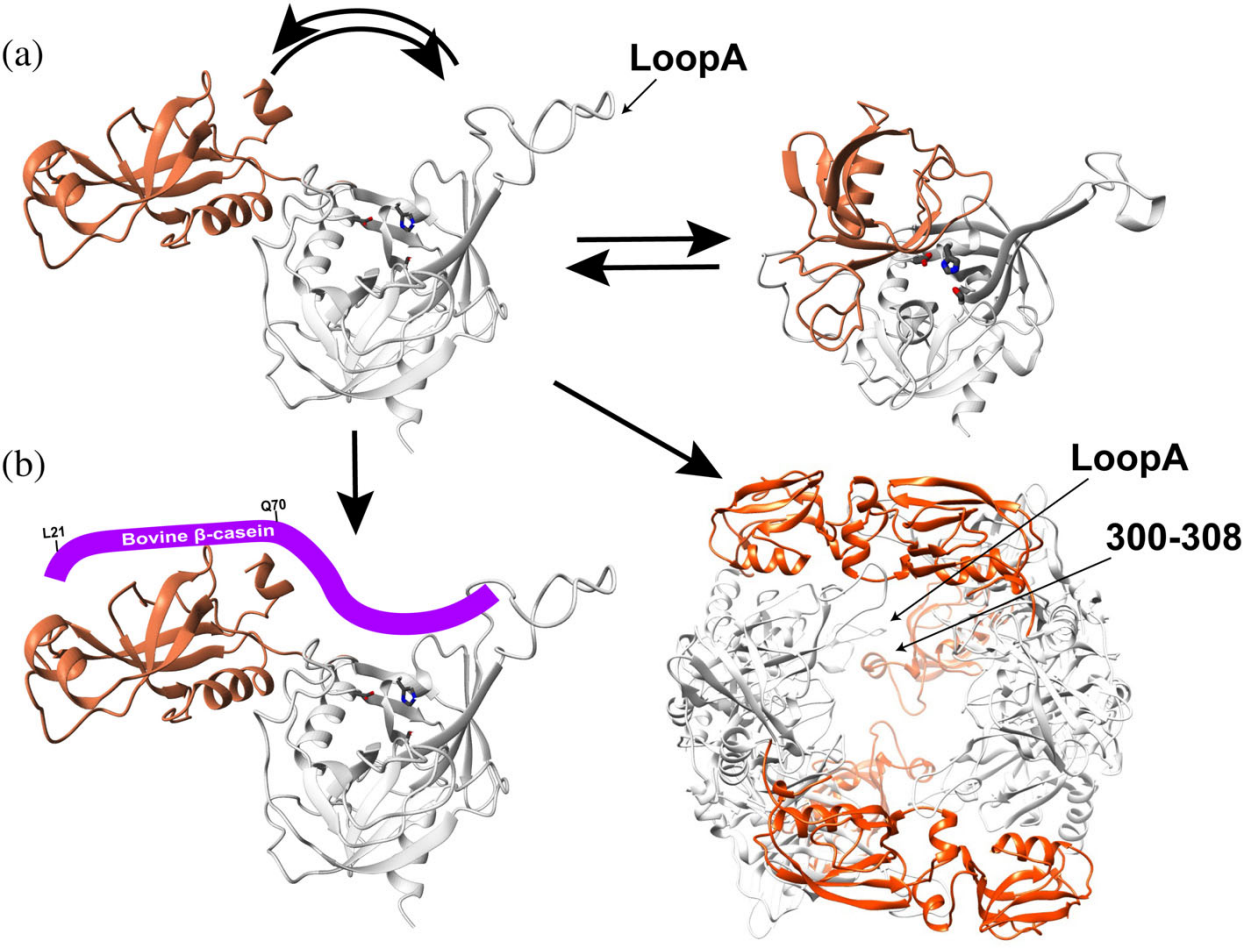
Proposed model of *S. pneumoniae* HtrA interactions. (a) *S. pneumoniae* HtrA dynamically samples both open and closed conformations in solution. The closed conformation was modeled using Swiss‐Model based on the structure of human HtrA2 (PDB ID 1ICY). (b) The HtrA monomer initially engages substrates such as β‐casein through the substrate N‐terminal residues 21–70 (left), but can assemble into higher‐order oligomers, including hexameric complexes, in the presence of specific substrates (right). The hexameric complex represents that from the *S. pneumoniae* HtrA S234A mutant with β‐casein.

Our NMR data reveal that *S. pneumoniae* HtrA samples at least two conformational states in solution. Chemical shift perturbations from titration experiments show that several resonances in the full ectodomain, particularly near Loop A, lie between those of the independently folded PD and the PD saturated with the isolated PDZ domain—consistent with a dynamic equilibrium between open and closed forms. R_2_‐CPMG relaxation dispersion experiments further support an inherent μs–ms exchange present only within the full ectodomain, but not in the isolated PD or the PD biochemically saturated with PDZ. This suggests an intrinsic flexibility that likely facilitates PD–PDZ gating. Although regulatory coupling between the PD and PDZ varies across HtrA family members, there is precedent for similar behavior. In human HtrA2, a “closed” state has been captured by X‐ray crystallography in which the PDZ sterically occludes the protease active site (Li et al., 2002), consistent with a gating mechanism in which the PDZ alternates between accessible and occluding conformations. By contrast, in *E. coli* DegS, the PDZ allosterically stabilizes an inactive protease conformation, and binding of hydrophobic peptides to the PDZ relieves this inhibition (Wilken et al., 2004). The dynamic equilibrium we observe in *S. pneumoniae* HtrA may be consistent with the human HtrA2‐type gating mechanism, with transient sampling modeled here using the HtrA2 structure (Figure 10a). Such PDZ‐mediated gating may represent a conserved regulatory strategy among diverse HtrA proteases.

Efforts to structurally characterize direct PD–PDZ interactions using intermolecular NOEs between purified domains were unsuccessful, likely due to transient or weak contacts. However, future alternative methods could potentially be used to further explore such phenomena. For example, FRET‐based methods with various labels could monitor distances in real time or hydrogen–deuterium exchange mass spectrometry could be used to identify protected regions.

### 3.3 PDZ‐dependent activation and a novel hexamer interface

Our results demonstrate that the PDZ domain of *S. pneumoniae* HtrA is required for proteolytic activity. This aligns with findings from other single‐PDZ HtrAs such as *M. tuberculosis* HtrA (PepD), which similarly require the PDZ domain for function (Gupta et al., 2018). In contrast, *E. coli* DegS retains proteolytic activity in the absence of its PDZ, highlighting functional divergence within the HtrA family (Wilken et al., 2004).

Notably, we directly demonstrate that *S. pneumoniae* HtrA requires its PDZ domain for substrate cleavage, reinforcing its essential role in protease function. This finding is consistent with our NMR titration experiments, as well as previous studies showing that the PDZ domain of *S. pneumoniae* HtrA binds short hydrophobic peptides (Fan et al., 2011). Although the functional consequences of peptide binding are difficult to quantify due to the low in vitro protease activity, the cumulative evidence supports a critical role for the PDZ domain in substrate engagement and recognition.

Our combined application of NMR and cryo‐EM has revealed two distinct oligomeric states of the catalytically inactive S234A mutant that could be separated by size‐exclusion chromatography. These include a monomeric form, where allosteric effects emanating from the mutation site could be quantified in solution, and a hexameric assembly resolved via cryo‐EM single particle reconstruction. NMR studies demonstrated long‐range chemical shift perturbations throughout the protein that could be tracked by CSPs, consistent with a conformational relay linking the active site to much of the PD and even the PDZ domain. This allosteric responsiveness may serve as a regulatory precondition for the observed hexamer formation. For example, *S. pneumoniae* HtrA may form hexameric assemblies in response to specific substrates, while the S234A mutation may simply predispose this assembly that could then be slow to disassemble. In this scenario, endogenous peptides from *E. coli* expression could either have induced hexamer formation and/or remained bound during purification. Co‐purified peptides have been observed for several other HtrAs, including DegQ (Krojer, Pangerl, et al., 2008; Sawa et al., 2011) and DegS (Wilken et al., 2004), where they contributed to PDZ‐mediated stabilization. Thus, while it remains unclear whether the *S. pneumoniae* WT HtrA samples such oligomeric states under physiological conditions, the fact that a single point mutation can drive a cooperative transition into a hexamer suggests the existence of a biologically relevant oligomerization pathway. However, caution must be taken to definitively conclude that such larger assemblies occur for the WT HtrA, as we have yet to identify such a hexamer for the *S. pneumoniae* WT HtrA. Ongoing efforts aim to identify other substrates that might trigger this assembly in the native enzyme.

Cryo‐EM analysis of the S234A mutant revealed a hexamer stabilized by canonical intra‐trimer PD–PD contacts and a potentially unique inter‐trimer interface involving PD LoopA interacting with residues 300–308 in the PDZ domain of an adjacent trimer. Although the resolution in this region is limited, the possible presence of this contact suggests a potentially novel stabilization mechanism that may be transient or dependent on specific substrate interactions. Given the modest local resolution, this proposed LoopA–PDZ interface should be interpreted with caution and will require further validation through mutagenesis or higher‐resolution structural studies to confirm its precise nature. To our knowledge, this LoopA–PDZ interface has not been described in other HtrA structures and could represent a structural feature unique to Gram‐positive HtrAs (Figure 10b). For example, the only other known resting HtrA with a single PDZ is the Gram‐negative *Synechocystis* sp. HhoA (Hall et al., 2017). However, in HhoA, trimer–trimer stabilization occurs through symmetric PDZ–PDZ contacts, rather than the PD–PDZ interaction observed here for the *S. pneumoniae* HtrA S234A hexamer. In contrast to these single‐PDZ HtrAs, DegP, and DegQ, which each contain two PDZ domains, undergo substrate‐induced transitions from resting hexamers into 12‐ and 24‐mer cage‐like oligomers, a process similarly driven by PDZ–PDZ interactions like the HhoA hexamer (Krojer et al., 2010; Malet et al., 2012; Sawa et al., 2011). Thus, the architecture observed in *S. pneumoniae* HtrA may define a new oligomeric interface distinct from previously characterized HtrA assembly modes.

## 4 CONCLUSIONS

The architecture observed in *S. pneumoniae* HtrA defines a new oligomeric interface distinct from previously characterized HtrA assembly modes. More broadly, these findings highlight that Gram‐positive HtrAs may constitute a mechanistically divergent subgroup within the larger HtrA family, relying on dynamic monomeric states and unique PD–PDZ interactions rather than stable trimeric cores. This divergence suggests alternative evolutionary solutions for coupling stress sensing to function and underscores the further need to elucidate interactions of Gram‐positive HtrAs in order to understand their roles in virulence.

## 5 MATERIALS AND METHODS

### 5.1 Bacterial strains and growth conditions

WT *S. pneumoniae* serotype 2 strain D39 (Wen et al., 2015) and the *ΔhtrA* knockout strain were cultured in Todd‐Hewitt broth supplemented with 0.5% yeast extract (THY) or on blood agar plates at 37°C in 5% CO_2_. For antibiotic selection, *ΔhtrA* strains were grown in THY supplemented with kanamycin (300 μg/mL).

The *ΔhtrA* strain was constructed using homologous recombination. Briefly, flanking regions (~0.5 kb) upstream and downstream of the *htrA* gene were PCR‐amplified from *S. pneumoniae* genomic DNA and ligated to a kanamycin resistance cassette from the Janus cassette. This recombinant construct was introduced into *S. pneumoniae* via natural transformation in minimal media containing competence‐stimulating peptides, and transformants were selected on blood agar plates containing kanamycin (300 μg/mL). Successful *htrA* deletion was confirmed by colony PCR and sequencing.

### 5.2 Adherence and murine infection assays

The role of HtrA in bacterial adhesion was assessed using A549 human alveolar epithelial cells and D562 human pharyngeal epithelial cells (obtained from the American Type Culture Collection). Cells cultured in Ham's F‐12K (Life Technologies Corporation) supplemented with 10% fetal bovine serum were seeded in 24‐well tissue culture plates at a density of 1 × 10^5^ cells per well and cultured overnight at 37°C in 5% CO_2_ until reaching confluence. *S. pneumoniae* strains were added at a multiplicity of infection (MOI) of 10 and plates were centrifuged for 3 min at 1000 × *g* followed by a 1 h incubation at 37°C in 5% CO_2_ to allow for adhesion. Following incubation, non‐adherent bacteria were removed by aspirating media and washing 3× with HBSS(−) and 1× with phosphate‐buffered saline (PBS). Host cells were lysed following trypsin–EDTA digestion for 15 min at 37°C in 5% CO_2_ by the addition of 300 μL sterile distilled water. The lysates were serially diluted and plated on blood agar to quantify colony‐forming units (CFUs). Percent adhesion was calculated relative to CFUs obtained from cell‐free media controls (input).

To assess the impact of HtrA on pneumococcal colonization and infection, C57BL/6J wild‐type mice (*n* = 5 per group, mixed‐sex) were infected intranasally under isoflurane anesthesia with 1 × 10^7^ CFU of WT or *ΔhtrA S. pneumoniae* in 50 μL PBS. At 96 h post‐infection, bacterial burdens were determined from the nasal lavage, bronchoalveolar lavage (BAL), and lung tissue. Lavage samples were collected by flushing the nasal cavity or lungs with sterile PBS, while lung tissue was homogenized in PBS using a bead mill homogenizer. Serial dilutions of each sample were plated on blood agar for CFU enumeration. The limit of detection (LOD) for bacterial quantification was 10 CFU per sample, and samples with undetectable CFUs were recorded as below the LOD.

All adherence and infection experiments were performed in triplicate and repeated a minimum of three times. Bacterial burdens were compared between groups using a Student's *t*‐test or Mann–Whitney *U*‐test, as appropriate, based on normality testing (GraphPad Prism). A *p*‐value of <0.05 was considered statistically significant. For in vivo experiments, power calculations determined that *n* = 5 per group would provide 80% power to detect a 0.5 log difference in CFUs with a 5% significance level.

### 5.3 Proteomic analysis of HtrA‐regulated proteins

To assess global proteomic changes associated with *S. pneumoniae* HtrA, wild‐type (WT) and *ΔhtrA* strains were cultured in 10 mL of THY medium to an optical density (OD₆₀₀) of ~1.0. Bacterial cultures were pelleted by centrifugation at 5000 × *g* for 10 min at 4°C, washed twice with PBS, and prepared for mass spectrometry using S‐Trap™ micro filters (Protifi, Huntington, NY) according to the manufacturer's protocol. Digested peptides were loaded onto individual Evotips and separated on an Evosep One chromatography system (Evosep, Odense, Denmark) using a Pepsep column, (150 μm inner diameter, 15 cm) packed with ReproSil C18 1.9 μm, 120A resin. The system was coupled to a timsTOF Pro mass spectrometer (Bruker Daltonics, Bremen, Germany) via the nano‐electrospray ion source (Captive Spray, Bruker Daltonics). The mass spectrometer was operated in PASEF mode. The ramp time was set to 100 ms and 10 PASEF MS/MS scans per topN acquisition cycle were acquired. MS and MS/MS spectra were recorded from *m*/*z* 100 to 1700. The ion mobility was scanned from 0.7 to 1.50 Vs/cm^2^. Precursors for data‐dependent acquisition were isolated within ±1 Th and fragmented with an ion mobility‐dependent collision energy, which was linearly increased from 20 to 59 eV in positive mode. Low‐abundance precursor ions with an intensity above a threshold of 500 counts but below a target value of 20,000 counts were repeatedly scheduled and otherwise dynamically excluded for 0.4 min. Data processing and statistical analysis of MS raw files were processed using MSFragger (FragPipe v19) against the *S. pneumoniae* D39 proteome database. Peptide‐spectrum matches (PSMs) were validated using Percolator with a 1% false discovery rate (FDR) at the protein level. Label‐free quantification was performed using IonQuant, and differentially expressed proteins were analyzed using MetaboAnalyst v5.0. Statistical significance was determined using *t*‐tests (two groups) or ANOVA (multiple groups) with FDR correction (*p* < 0.05).

### 5.4 Protein expression and purification

For the full ectodomain and PD of *S. pneumoniae* HtrA, residues 59–393 and residues 59–285 (uniprot accession number A0A0H2ZQD7), respectively, were both cloned with an N‐terminal 6xHis tag into pET21 and expressed in BL21(DE3) cells. For a typical 4 L growth, cells were grown in Luria Broth (LB) and induced with IPTG at an OD(600) of 0.6 for 3 h at 37°C. For either ^15^N labeling or ^15^N,^13^C‐labeling, an LB to minimal media switch was used, whereby 4 L were spun at 0.5 OD(600) to M9 minimal media either using ^15^N ammonia or both ^15^N ammonia and ^13^C‐glucose, respectively. Bacterial cells were harvested, sonicated in Ni A buffer (50 mM Na_3_PO_4_, pH 7, 500 mM NaCl, 10 mM imidazole), centrifuged, and supernatants passed over a Ni‐column (Sigma). Protein was eluted with Ni B (Ni A plus 400 mM imidazole), concentrated, and applied to a Sephadex‐200 preparative sizing column (Cytiva) equilibrated in NMR buffer (50 mM Na_2_PO_4_, pH 6, 50 mM NaCl). Additionally, for NMR assignments, both the full ectodomain and the PD were grown in 99% D_2_O and subsequently refolded after the initial Ni‐affinity column, as previously described (Paukovich et al., 2018).

For the *S. pneumoniae* HtrA PDZ domain, residues 285–493 were cloned downstream of human SUMO1 (hSUMO1) in order to produce the PDZ with no overhang. Expression proceeded as described above for the His‐tagged proteins, with the following changes to protein purification. Post Ni‐affinity, PDZ elutions were dialyzed back to Ni A, the protein cleaved by ULP1 (recombinantly produced in‐house from pFGET19_ULP1, Addgene), and the 6H‐hSUMO1 removed via Ni‐affinity. Fractions containing the PDZ were concentrated and applied to a Sephadex‐75 preparative sizing column equilibrated in NMR buffer.

For *E. faecium* HtrA (uniprot accession number J7CU12) and *S. aureus* HtrA1 (uniprot accession number A0A0H2XGV4), full ectodomains of residues 75–429 and 83–424, respectively, were cloned into pET21 and purified as the full ectodomain of *S. pneumoniae* HtrA.

For caseins, full‐length β‐casein and α‐casein were commercially purchased (Sigma), while all other constructs were recombinantly produced in‐house. Both β‐casein residues 21–70 and 71–130 were produced using the same hSUMO1 system described above for the HtrA PDZ. Human β‐casein and bovine β‐casein 101–224 were produced with an N‐terminal 6xHis‐tag and purified as described for the full HtrA ectodomain and PD.

### 5.5 NMR studies

All NMR samples were prepared in the defined NMR buffer supplemented with 5% D_2_O. Backbone assignments were performed using uniformly ^15^N/^13^C‐labeled PDZ and ^15^N/^13^C/2H‐labeled PD and the full ectodomain at a concentration of 500 μM. All assignment spectra were acquired at 35°C on a Bruker 600 MHz spectrometer equipped with a cryoprobe. Standard triple‐resonance experiments including HNCACB, CBCA(CO)NH, HNCO, and HN(CA)CO were used to assign backbone resonances for the HtrA PDZ. However, for both the HtrA full ectodomain and the PD, both HNCA and CBCA(CO)NH optimized for CB were collected. Spectra were processed using NMRPipe (Delaglio et al., 1995) and analyzed in CcpNMR Analysis (Vranken et al., 2005).

Titration experiments were conducted on a Bruker 900 MHz spectrometer equipped with a TCI cryoprobe at 35°C. ^15^N‐HSQC spectra were collected at each titration point with the indicated concentrations. Chemical shift perturbations (Δ*δ*) were calculated using the combined chemical shift difference formula: Δ*δ* = sqrt ((5*Δ*δ*H)^2^ + (Δ*δ*N)^2^), as previously reported (Williamson, 2013). The factor of five compensates for the smaller spectral dispersion for the ^1^H dimension. Titration curves were analyzed and fit to a single‐site binding model using GraphPad Prism. Spectra were processed with NMRPipe and visualized using CcpNMR Analysis.

### 5.6 Cryo‐EM sample preparation, data collection, and model building

For *S pneumoniae* HtrA, purified protein was concentrated to 183 μM in final storage buffer and used either alone (apo HtrA) or incubated with 810 μM β‐casein for the complex sample. Following incubation, the HtrA‐β‐casein complex was further separated by analytical sizing column. For cryo‐EM grid preparation, 3 μL of 0.3 mg/mL sample was applied to plasma‐cleaned C‐flat holey carbon grids (1.2/1.3, 400 mesh) and vitrified using a Vitrobot Mark IV (Thermo Fisher Scientific) at 100% humidity and 4°C. Grids were blotted for 3.0 s and flash‐frozen in liquid‐nitrogen‐cooled ethane.

A full description of cryo‐EM data collection is provided in Figures S2, S3, and Table S2. Both the apo and β‐casein‐bound HtrA samples were imaged on a Titan Krios microscope at the Pacific Northwest Center for Cryo‐EM. Data were processed in cryoSPARC (Punjani et al., 2017), with motion correction performed using MotionCor2 (Iverson et al., 2020) and CTF estimation carried out in Gctf (Zhang, 2016). Class averages were used for template‐based particle picking. Particles were extracted with a box size of 280 pixels and subjected to 2D classification. Well‐defined classes were selected for ab initio reconstruction, followed by 3D classification and non‐uniform refinement. Final map resolutions were determined using the gold‐standard Fourier shell correlation (FSC) criterion.

Initial models were built in Chimera (Pettersen et al., 2004), using homology‐based models generated from AlphaFold or related bacterial HtrA structures as references. Models were iteratively refined in Coot (Emsley et al., 2010) and Phenix (Adams et al., 2010).

### 5.7 Protein crystallization, crystallographic data collection, and structure refinement

The purified HtrA PDZ domain first was passed over a Bio‐Gel P‐6DG desalting column (BioRad; Hercules, CA, USA) equilibrated with 25 mM HEPES, pH 7.5 to remove salt from the storage buffer. High‐throughput crystallization trials for the HtrA PDZ domain were then carried out with commercially available screens in small‐volume sitting‐drop trays using a Crystal Gryphon LCP robot (Art Robbins Instruments; Sunnyvale, CA, USA). Drops consisting of 0.2 μL protein sample at 15 mg/mL were mixed with 0.2 μL reservoir solution and left to equilibrate at room temperature for a few weeks. The reservoir volume was 30 μL. Initial hits grew after 1–2 weeks in 0.05 M ammonium sulfate, 0.05 M Bis‐Tris, pH 6.5, and 30% (v/v) pentaerythritol ethoxylate (15/4_EO/OH) (MCSG1 F9) (Fazio et al., 2014). Crystals were taken directly from the small‐volume screening plates and cryoprotected with a mixture containing 80% (v/v) of the above reservoir solution supplemented with 20% (v/v) glycerol.

Diffraction data for the HtrA PDZ crystals were collected at the Canadian Light Source BM beamline on a Detectris Pilatus3 S 6M. Data were collected at a wavelength of 1.1807 Å with an exposure of 0.2°/0.2 s/frame. All data were indexed, integrated, and scaled with DIALS (version 3.8.0) (Winter et al., 2018) and imported into the CCP4i suite (version 8.0.009) (Winn et al., 2011) with AIMLESS (version 0.7.9) (Evans & Murshudov, 2013). Phases were solved using molecular replacement (MOLREP version 11.9.02) (Vagin & Teplyakov, 2010) with its corresponding AlphaFold2 model (UniProt Accession #A0A4J2AIC8; residues 285–393) (Jumper et al., 2021; Mirdita et al., 2022). Refinement was done using phenix.refine (version 1.20.1_4487) (Afonine et al., 2012) in conjunction with manual model building in COOT (version 0.8.9.2) (Emsley et al., 2010). B‐factors were refined isotropically with automatically determined translation‐libration‐screw parameters. Alternate conformer occupancies were also refined with phenix.refine (Afonine et al., 2012). The ISOLDE plugin (version 1.8) (Croll, 2018) in ChimeraX (version 1.8) (Meng et al., 2023) was used during early refinement to improve model quality. Final model geometry was analyzed and optimized based on suggestions by MolProbity (version 4.5.2) (Williams et al., 2018). Data collection and model statistics for all structures are summarized in Table S1.

## AUTHOR CONTRIBUTIONS

**Eunjeong Lee:** Conceptualization; investigation; formal analysis. **Jasmina S. Redzic:** Conceptualization; investigation; methodology; formal analysis. **Blaine Gordon:** Writing – original draft. **Anthony J. Saviola:** Investigation. **Norman Tran:** Investigation. **Sean P. Maroney:** Investigation. **Nathanael L. Ashby:** Investigation. **Steven Shaw:** Investigation. **Sam Fulte:** Investigation. **Arianna McCarty:** Investigation. **Todd Holyoak:** Investigation. **Nancy Meyer:** Investigation. **Kirk C. Hansen:** Investigation. **Sarah E. Clark:** Investigation. **Elan Eisenmesser:** Conceptualization; investigation; funding acquisition; methodology; formal analysis.

## CONFLICT OF INTEREST STATEMENT

The authors declare no conflict of interest.

## Supporting information


**Appendix S1:** Supporting information.

## Data Availability

The NMR chemical shift assignments for *S. pneumoniae* HtrA have been deposited in the Biological Magnetic Resonance Data Bank under accession numbers BMRB: 53293 (HtrA full ectodomain, residues 59–393), BMRB: 53294 (HtrA PD, residues 59–285), and BMRB: 53295 (HtrA PDZ, residues 285–393). The crystal structure of the HtrA PDZ domain is deposited in the Protein Data Bank under accession number PDB: 9PNO. The cryo‐EM structures of apo and substrate‐bound HtrA S234A hexamers have been deposited in the Protein Data Bank under accession numbers PDB: 9PU7 (apo HtrA) and PDB: 9PU4 (HtrA bound to bovine β‐casein), with corresponding EMDB accession numbers EMD‐71870 and EMD‐71866, respectively.
