# Supplementary material for: Streptococcus pneumoniae HtrA is a dynamic and monomeric virulence factor capable of forming larger oligomeric complexes
Source: Protein Sci. 2025 Dec 28;35(1):e70411. doi: 10.1002/pro.70411 (PMC12745498; doi:10.1002/pro.70411)
Supplement: Supplementary file 1 — Appendix S1: Supporting information. [file PRO-35-e70411-s001.doc]

**SUPPLEMENTARY MATERIAL**

**Title: *Streptococcus pneumoniae* HtrA is a dynamic and monomeric virulence factor capable of forming larger oligomeric complexes.**

Eunjeong Lee^1^, Jasmina S. Redzic^1^, Anthony J. Saviola^1^, Norman Tran^2^, Sean P. Maroney^1^, Nathanael L. Ashby^1^, Steven Shaw^3^, Sam Fulte^3^, Arianna McCarty^3^, Todd Holyoak^2^, Nancy Meyer^4^, Kirk C. Hansen^1^, Sarah E. Clark^3^, and Elan Eisenmesser^1†^.

^1^Department of Biochemistry and Molecular Genetics, School of Medicine, University of Colorado Anschutz Medical Campus, School of Medicine, Aurora, CO 80045

^2^Department of Biology, University of Waterloo, Waterloo ON, Canada N2L 3G1

^3^Department of Otolaryngology – Head & Neck Surgery, School of Medicine, University of Colorado Anschutz Medical Campus, School of Medicine, Aurora, CO 80045

^4^Pacific Northwest Cryo-EM Center, Oregon Health and Science University, Portland, OR 97201

^†^Corresponding author: Elan.Eisenmesser@ucdenver.edu

Contents:

Figure S1

Figure S2

Figure S3

Figure S4

Figure S5

Figure S6

Tables

Table S1

Table S2


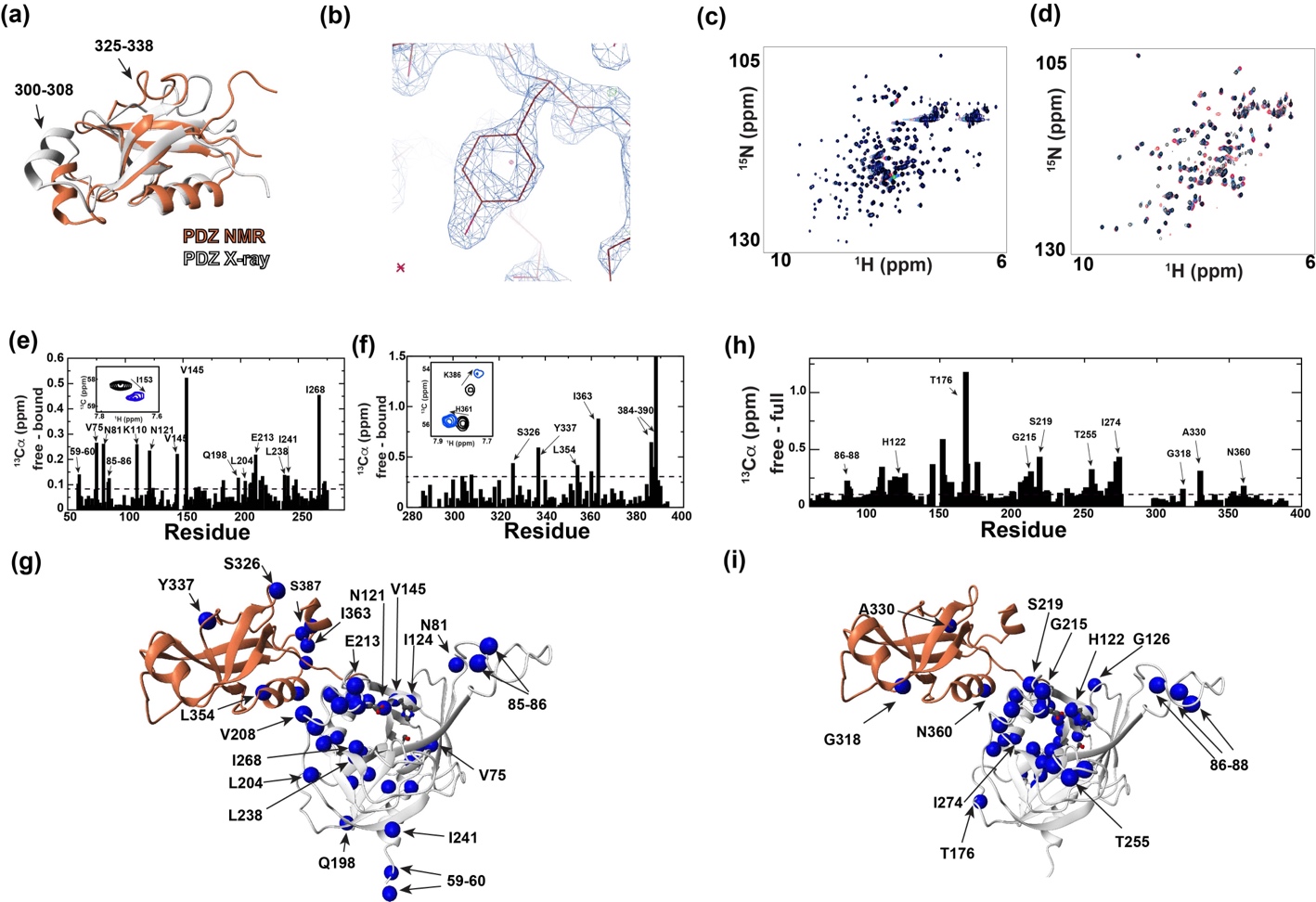


**Figure S1. Structural studies of *S. pneumoniae* HtrA and its domain interactions.**

(a) X-ray crystal structure of the PDZ (white) compared to the NMR structure (orange, PDB ID 2l97). Two regions that exhibit differences between the two structural models are delineated and include residues 300-308 and 325-338,

(b) Sample density of the PDZ at 2F_o_-F_c_ electron density of 0.9σ.

(c) Full spectra of ^15^N-labeled PD titrated with unlabeled PDZ Spectra include the following: free (black), 0.25 mM (blue), 0.5 mM (sky blue), 1 mM (green), 2 mM (mauve), and 4 mM (red).

(d) Full spectra of ^15^N-labeled PDZ titrated with unlabeled PD. Spectra include the following: free (black), 0.25 mM, 0.5 mM (sky blue), 1 mM (green), 2 mM (mauve), and 4 mM (red).

(e) Absolute Cα differences between the HtrA PD and the same residues within the context of the full ectodomain, derived from 3D HNCA spectra (600 MHz) with the inset illustrating one such example. The average was 0.05 ppm and the standard deviation was 0.063 ppm. The average plus a half standard deviation of 0.083 ppm is delineated (dashed line).

(f) Absolute Cα differences between the HtrA PDZ alone and in the presence of 4 mM PD, derived from 3D HNCA spectra (600 MHz) with the inset illustrating one such example. The average was 0.15 ppm and the standard deviation was 0.20 ppm. The average plus a half standard deviation of 0.25 ppm is delineated (dashed line).

(g) Cα CSPs larger than the average plus a half standard deviation are mapped onto the HtrA model (blue spheres) from (e) and (f).

(h) Amide differences between the free HtrA domains and within the context of the full HtrA construct residues 59-393. The average was 0.098 ppm and the standard deviation was 0.011 ppm. The average plus a half standard deviation of 0.153 ppm is delineated (dashed line).

(i) Amide CSPs larger than the average plus a half standard deviation are mapped onto the HtrA model (blue spheres) from (h).


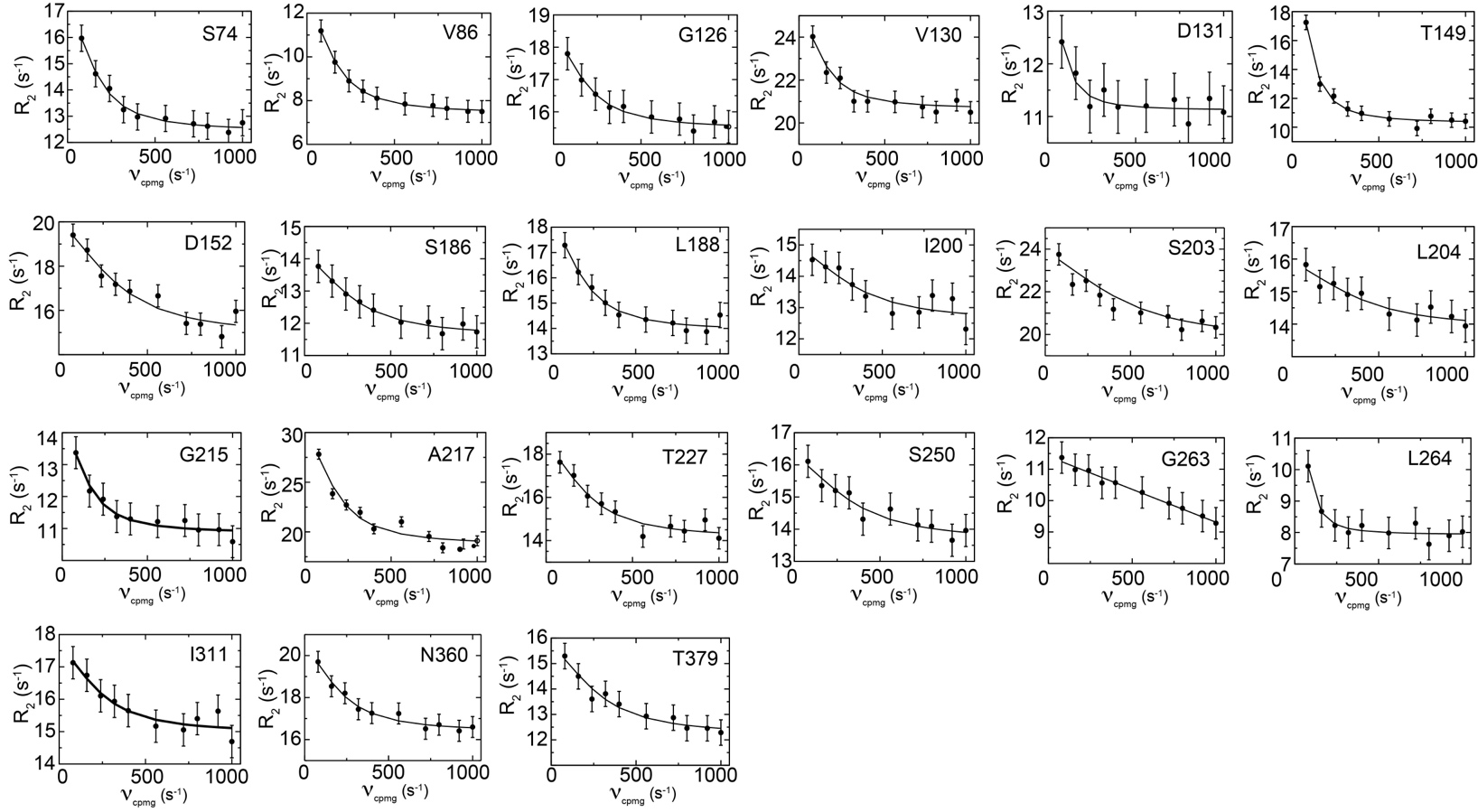


**Figure S2.** R_2_**-CPMG dispersion profiles for *S. pneumoniae* HtrA residues 59-393.** Full R_2_-CPMG dispersion profiles are shown for a global fit of HtrA residues 59-393, except for G263 and L264 that exhibited faster and slower fit exchange rates, respectively. The extracted global exchange rate was 950 ppm ± 200 s^-1^ while the minor populations were poorly defined and could be well fit using 30-45%. No dispersions were observed for either HtrA PD alone or saturated with 4 mM PDZ.

**
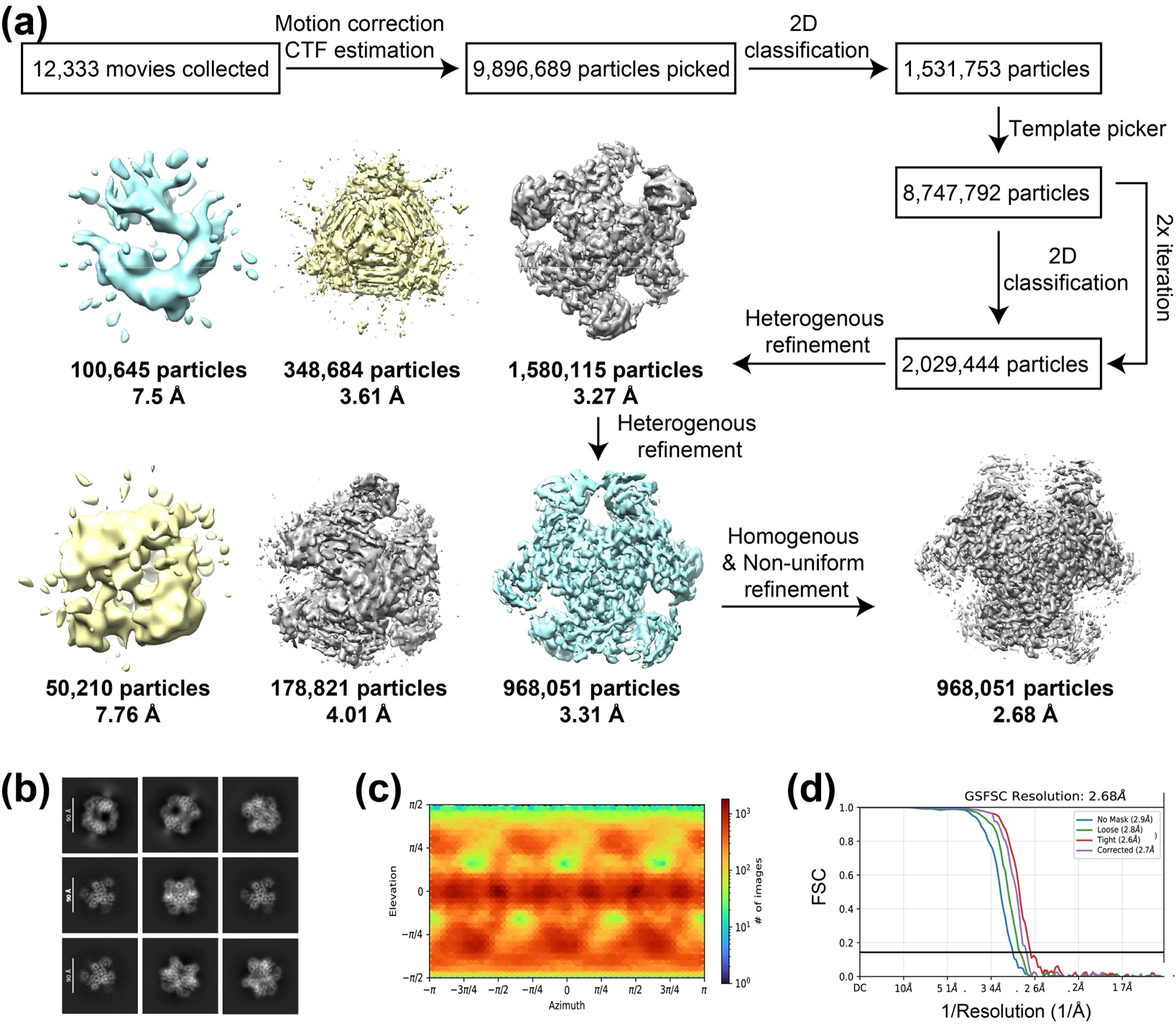
**

**Figure S3. Workflow for cryo-EM data processing for the apo *S. pneumoniae* HtrA H234A hexamer.**

(a) Overall workflow of cryo-EM data processing with specific examples and FSC resolutions.

(b) Representative 2D class averages.

(c) Angular distribution heat map of final particle projections.

(d) Fourier shell correlation (FSC) curves.

All data were processed in CryoSPARC.

**
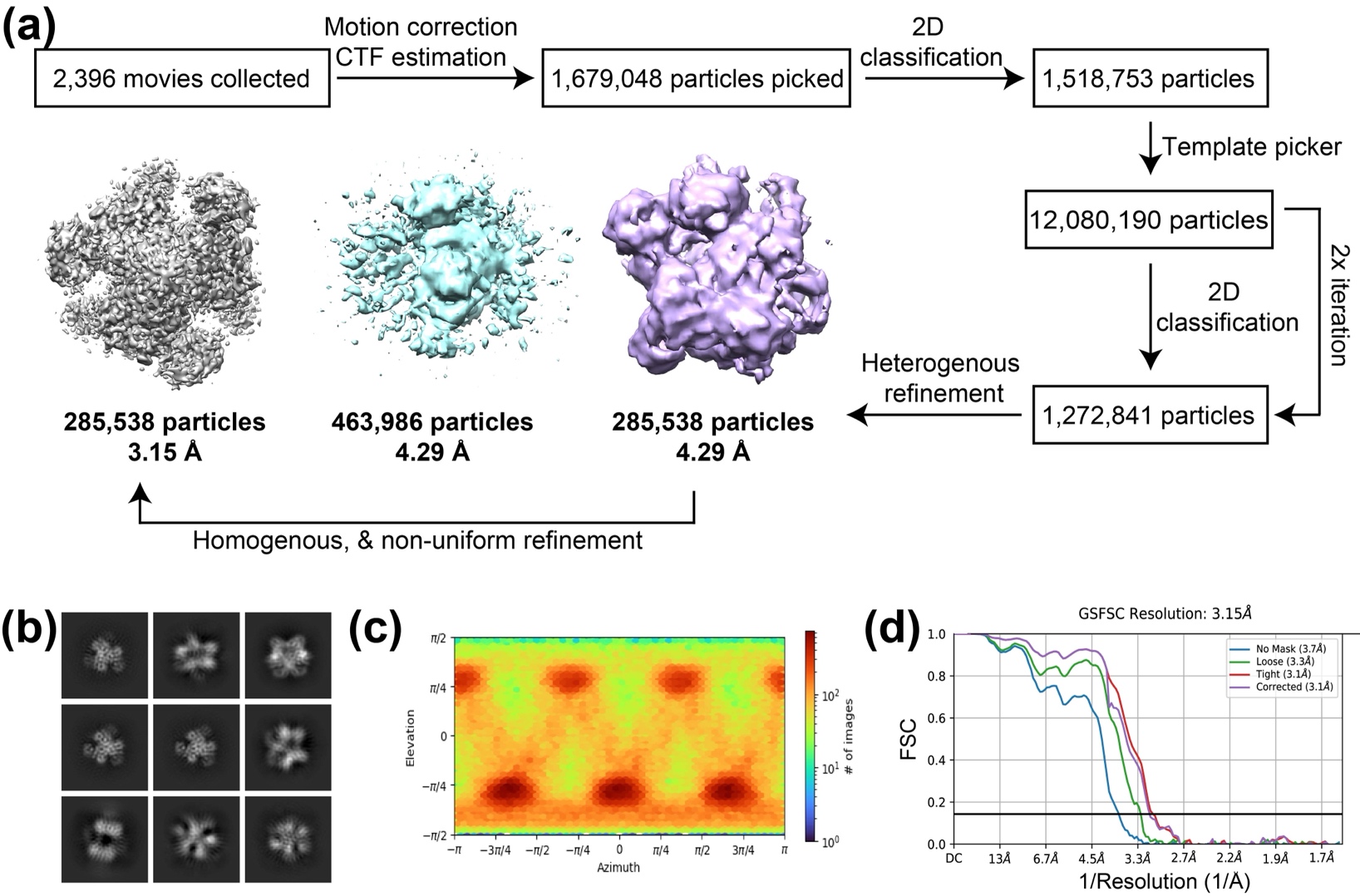
**

**Figure S4. Workflow for cryo-EM data processing for the *S. pneumoniae* HtrA H234A/β-casein complex hexamer.**

(a) Overall workflow of cryo-EM data processing with specific examples and FSC resolutions.

(b) Representative 2D class averages.

(c) Angular distribution heat map of final particle projections.

(d) Fourier shell correlation (FSC) curves.

All data were processed in CryoSPARC.

**
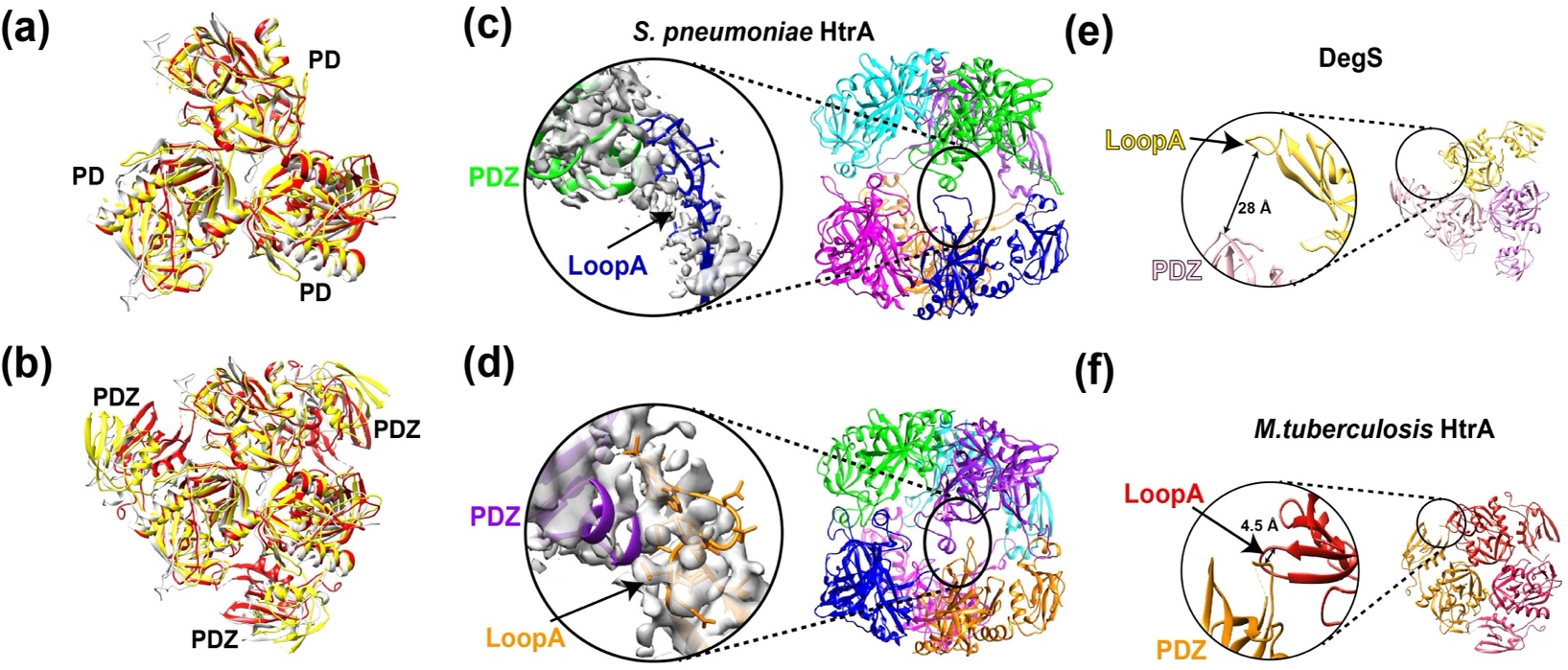
**

**Figure S5.** **Structural comparisons of Loop A conformations and PDZ interactions.**

(a) PD trimer superposition of *S. pneumoniae* HtrA (white), the E. coli DegS (yellow), and M. tuberculosis HtrA (red).

(b) Full HtrA superposition of *S. pneumoniae* HtrA (white), the E. coli DegS (yellow), and M. tuberculosis HtrA (red) with the PDZ indicated.

(c) Structural model of the apo S. pneumoniae HtrA 234A hexamer illustrating trimer/trimer interactions with ablow-up view with density highlighting a dynamic LoopA/PDZ interaction. PDZ and LoopA density were to low to accurately model the explicit interactions.

(d) Structural model of the S. pneumoniae HtrA 234A/β-casein complex hexamer illustrating trimer/trimer interactions with a blow-up view with density highlighting a dynamic LoopA/PDZ interaction. PDZ and LoopA density were to low to accurately model the explicit interactions.

(e) Structure of trimeric the E. coli DegS highlighting LoopA and the PDZ domain illustrating no interaction (PDB ID 4RR1).

(f) Structure of trimeric the M. tuberculosis HtrA highlighting a LoopA interaction with neighboring PDZ domain (PDB ID 7W4U).

**
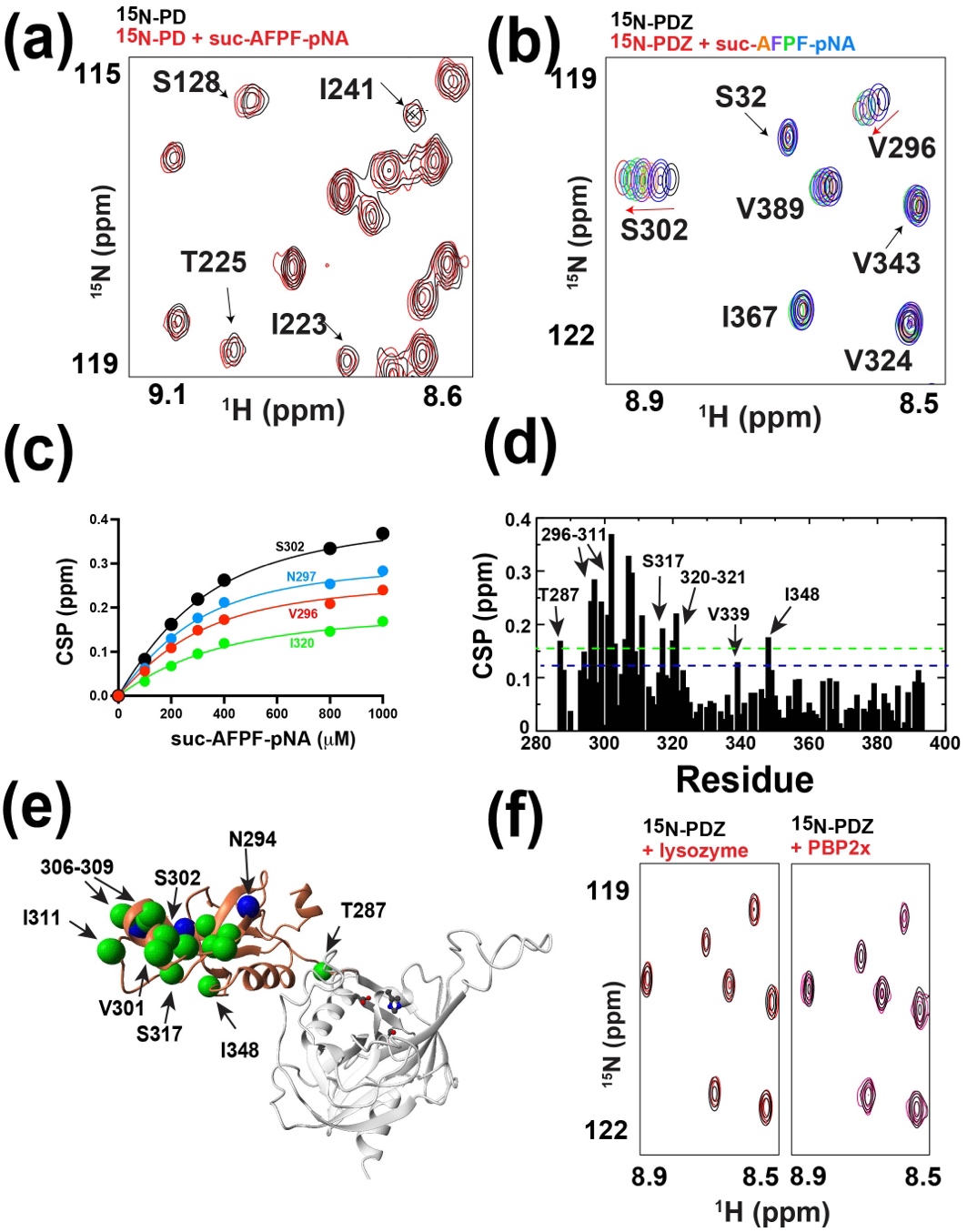
**

**Figure S6. *S. pneumoniae* HtrA domain specific interactions with a hydrophobic peptide.**

(a) Selected region of a ^15^N-HSQC for 250 μM HtrA PD alone (black) and in the presence of 500 μM suc-AFPF-pNA (red).

(b) Selected region of a ^15^N-HSQC for 250 μM HtrA PDZ alone (black) and in the presence of 500 μ suc-AFPF-pNA (red).

(c) Binding isotherms of ^15^N-PDZ from titrations with suc-AFPF-pNA are shown that were globally fit with an extracted KD of 150 ± 30 μM. Only a subset of the nearly two dozen amide shifts are shown for clarity.

(d) Amide CSPs between ^15^N-PDZ alone and in the context of 500 μM suc-AFPF-pNA. The average CSP was 0.09 ppm ± 0.07 ppm standard deviation. Dashed lines correspond to the average plus one standard deviation (0.16 ppm, green) and ½ standard deviation (0.13 ppm, blue).

(e) CSPs are mapped onto the HtrA PDZ within the model structure with spheres color-coded and described in (d).

(f) Selected regions of a ^15^N-HSQC for 250 μM HtrA PDZ alone (black) and in the presence of either lysozyme (red, left) and PBP-2x (red, right).

**
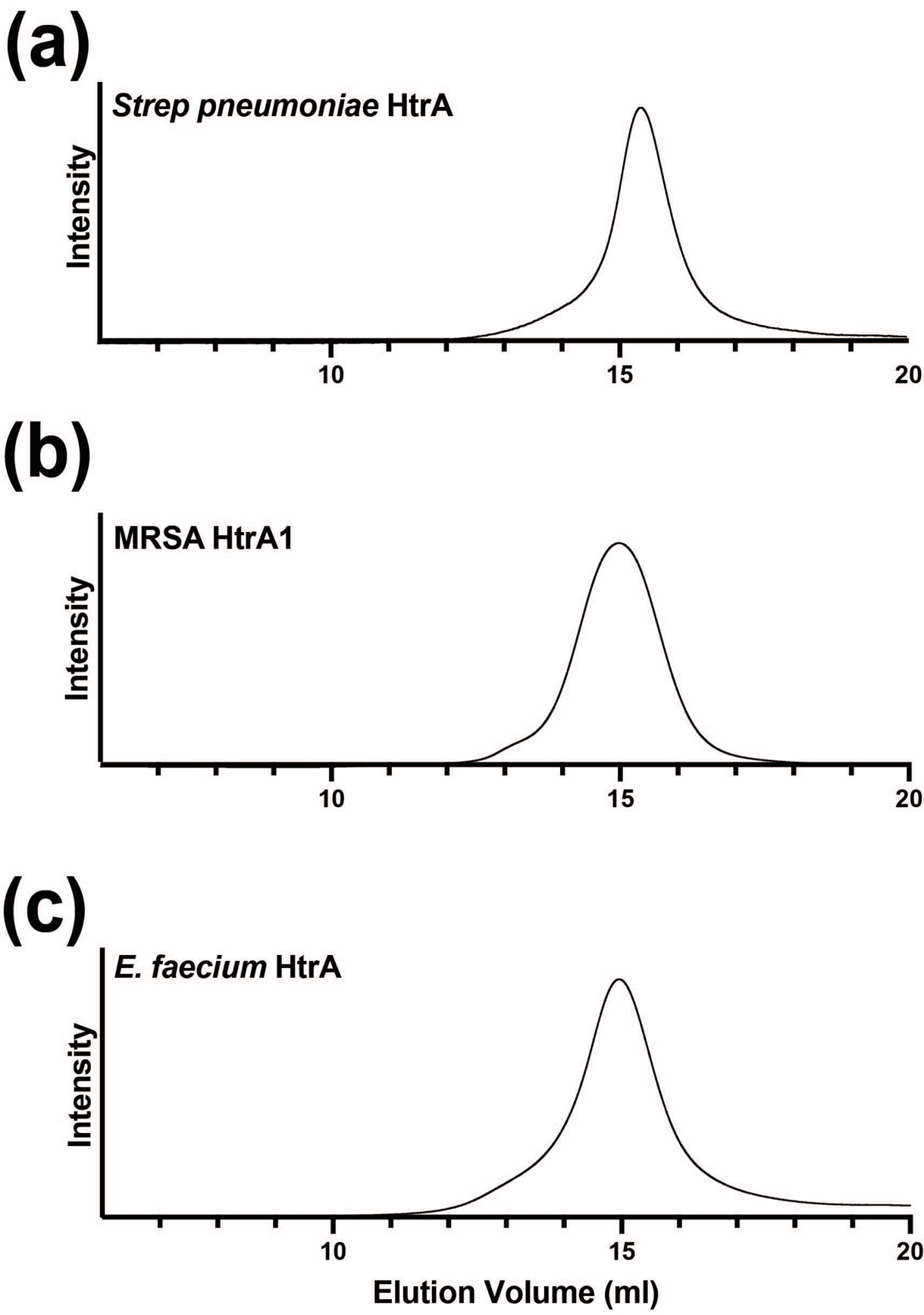
**

**Figure S7. Analytical size-exclusion chromatography reveals that HtrA proteins from other Gram-positive bacteria are monomeric.**

(a) Elution profile of *S. pneumoniae* HtrA.

(b) Elution profile of *E. faecium* HtrA.

(c) Elution profile of *S. aureus* HtrA1.

Proteins were run on an analytical Superdex-200 at 4°C. Elution volumes were consistent with monomeric species.

**Table S1.** X-ray crystallography data and refinement statistics for the HtrA PDZ domain crystallographic dataset. Statistics for the highest-resolution bin are in parentheses.

| Wavelength (Å) | 1.1807 |
| --- | --- |
| Resolution range (Å) | 61.50 – 1.85 (1.88 – 1.85) |
| Space group | P2_1_2_1_2_1_ |
| Unit cell (Å) | 31.99 50.09 122.99 |
| Total reflections | 210783 (10268) |
| Unique reflections | 17548 (859) |
| Multiplicity | 12.01 (11.95) |
| Completeness (%) | 99.73 (98.01) |
| Mean I/s(I) | 4.2 (0.2) |
| Wilson B-factor (Å^2^) | 26.30 |
| R_merge_ | 0.173 (0.950) |
| R_meas_ | 0.181 (0.993) |
| R_pim_ | 0.052 (0.285) |
| CC_1/2_ | 0.996 (0.604) |
| Number of reflections used in refinement | 17494 (833) |
| Number of reflections used for R_free_ | 851 (37) |
| R_work_ | 0.216 (0.332) |
| R_free_ | 0.248 (0.466) |
| Molecules per asymmetric unit | 2 |
| Number of atoms | 1822 |
| Protein | 1655 |
| Ligands | 20 |
| Water | 147 |
| Bond RMSD (Å) | 0.003 |
| Angle RMSD (^o^) | 0.51 |
| Ramachandran (%) |  |
| Favored | 97.63 |
| Allowed | 2.37 |
| Outliers | 0 |
| Rotamer outliers (%) | 0.0 |
| Clashscore (percentile) | 5.05 (96^th^) |
| MolProbity score (percentile) | 1.34 (98^th^) |
| PDB accession code | 9PNO |

**Table S2**. Cryo-EM data collection and model refinement of Apo HtrA 234A, HtrA 234A complex with β-casein.

|  | Apo HtrA 234A | HtrA 234A with β-casein |
| --- | --- | --- |
| Data Collection and Processing | | |
| Microscope | Titan Krios | Titan Krios |
| Voltage (kV) | 300 | 300 |
| Magnification | 29,000 | 29,000 |
| Electron Dose (e-/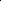^2^) | 48 | 48 |
| Camera | Gatan K3 | Gatan K3 |
| Defocus range (um) | -0.8 to -2.3 | -0.8 to -2.2 |
| Pixel size (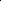) super resolution | 0.7296 | 0.394 |
| Movies collected | 13,596 | 1,498 |
| Symmetry imposed | D3 | C3 |
| Final particle images (no.) | 968,051 | 285,538 |
| Map resolution (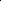) | 2.68 | 3.15 |
| Sharpening B-factor (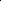^2^) | 150.17 | 111.21 |
| Software used to process data | cryoSPARC | cryoSPARC |
| Refinement statistics |  |  |
| Number of protein atoms (non-H) | 14850 | 14850 |
| R.m.s deviations |  |  |
| Bond lengths (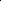) | 0.009 | 0.01 |
| Bond angles (°) | 0.889 | 1.094 |
| Validation |  |  |
| MolProbity score | 2.28 | 2.80 |
| Clash score | 15.43 | 34.36 |
| Poor rotamers (%) | 0 | 0.23 |
| Ramanchandram plot |  |  |
| Favored (%) | 88.69 | 76.28 |
| Allowed (%) | 11.01 | 23.57 |
| Disallowed (%) | 0.3 | 0.15 |
| Model vs Data CC | 2.5 | 3.5 |
| FSC model (0.143) | 2.8 | 3.1 |
| EMDB accession code | D_1000298447 | D_1000298443 |
| PDB accession code | 9PU7 | 9PU4 |
